# TALDO1 promotes lipid metabolic reprogramming and immunosuppressive microenvironment remodelling in hepatocellular carcinoma

**DOI:** 10.1002/ctm2.70771

**Published:** 2026-08-10

**Authors:** Fenglin Lv, Huaxin Zhou, Jingyan Yang, Jianglei Xu, Fanqing Bi, Yi Zhou, Hao Zhang, Qian Ye, Lin Gao, Bin Jin

**Affiliations:** ^1^ Department of Hepatobiliary Surgery The Second Qilu Hospital of Shandong University Jinan China; ^2^ Shandong Province Engineering Research Center for Multidisciplinary Research on Hepatobiliary and Pancreatic Malignant Tumors Jinan China; ^3^ Department of Pathology The Second Qilu Hospital of Shandong University Jinan Shandong China; ^4^ The Department of Cardiovascular Ultrasound Central hospital affiliated to Shandong First Medical University Jinan China; ^5^ Department of General Surgery Qingdao Central Hospital School of Clinical Medicine University of Health and Rehabilitation Sciences Qingdao China; ^6^ Department of Infectious Diseases and Hepatology The Second Qilu Hospital of Shandong University Jinan China

**Keywords:** hepatocellular carcinoma, immunosuppression, lipid metabolic reprogramming, multi‐omics, single‐cell RNA sequencing, tumour microenvironment

## Abstract

**Background:**

Hepatocellular carcinoma (HCC) is characterized by pronounced metabolic reprogramming and is frequently accompanied by the development of an immunosuppressive microenvironment. However, the key molecular mediators linking tumour metabolic dysregulation to immune microenvironment remodelling remain insufficiently defined. This study aimed to identify critical metabolic genes in HCC and to investigate their roles in lipid metabolic reprogramming and immunosuppression.

**Methods:**

A deep autoencoder was used to extract latent metabolic features from HCC and identify TALDO1 as a key candidate gene. The expression pattern and prognostic significance of TALDO1 were evaluated across multiple independent cohorts and further validated in clinical specimens. Multi‐omics analyses combined with experimental validation were then used to elucidate the role of TALDO1 in lipid metabolic reprogramming and its effects on immune microenvironment remodelling in HCC.

**Results:**

TALDO1 was identified as a key metabolic gene associated with HCC progression and was consistently upregulated across multiple clinical cohorts. Mechanistically, TALDO1 promoted lipogenesis and lipid accumulation in HCC cells by suppressing AMPK activation and sustaining SREBP1 maturation. TALDO1 silencing also reduced the secretion of several fatty acids and lipid mediators. Multi‐omics analyses together with multiplex immunofluorescence validation showed that high TALDO1 expression was associated with an immunosuppressive microenvironment in HCC. Coculture experiments further demonstrated that TALDO1 silencing attenuated the ability of HCC cells to induce M2‐like macrophage polarization and promote phenotypes associated with T‐cell exhaustion. In vivo, TALDO1 loss was accompanied by reduced immunosuppressive cell infiltration, enhanced effector T‐cell activity, and impaired tumour growth. Consistently, TALDO1 silencing also markedly suppressed tumour growth in patient‐derived xenograft models.

**Conclusions:**

TALDO1 promotes lipid metabolic reprogramming in HCC and participates in the formation of an immunosuppressive microenvironment. These findings suggest that TALDO1 may serve as a key molecule linking metabolic abnormalities to immune microenvironment remodelling and may have potential therapeutic significance.

**Key points:**

- TALDO1 promotes lipid metabolic reprogramming in HCC by regulating AMPK/SREBP1 signaling.
- TALDO1 is associated with the formation of an immunosuppressive tumour microenvironment.
- Multi‐omics analyses identified an association of TALDO1 with HCC progression and poor prognosis.

## Graphical abstract

Deep autoencoder algorithm identified TALDO1 as a key metabolic gene associated with HCC progression. TALDO1 promotes lipid synthesis and accumulation in HCC cells and participates in lipid metabolic reprogramming. TALDO1 is associated with immunosuppressive microenvironment remodelling, and targeting TALDO1 reduces the infiltration of immunosuppressive cells in HCC.

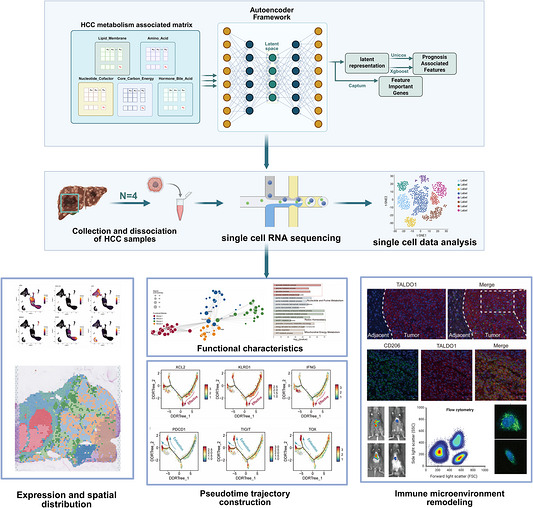

## 1 INTRODUCTION

Hepatocellular carcinoma (HCC) is one of the most common types of primary liver cancer and a major cause of cancer‐related death.^1^, ^2^, ^3^ Despite continuous advances in targeted therapy and immunotherapy in recent years, the long‐term prognosis of patients with HCC remains poor.^4^, ^5^ Therefore, further elucidating the key molecular mechanisms that drive HCC progression and identifying potential therapeutic targets remain major priorities in current research.

Metabolic reprogramming is a hallmark of cancer and is particularly prominent in HCC.^6^, ^7^, ^8^ During hepatocarcinogenesis and tumour progression, tumour cells often undergo extensive metabolic remodelling to adapt to the survival pressures imposed by sustained proliferation and a complex microenvironment.^9^, ^10^ Previous studies have shown that aberrant activation of multiple metabolic pathways in HCC is closely associated with poor prognosis and therapeutic resistance.^9^ However, because metabolic networks are intrinsically highly complex and nonlinear, conventional single‐gene‐based analytical strategies often fail to accurately identify key metabolic genes from high‐dimensional transcriptomic data. At the same time, metabolic reprogramming not only determines the biological behaviour of tumour cells themselves, but also affects immune cell function through metabolite accumulation and intercellular signalling, thereby shaping an immunosuppressive tumour microenvironment (TME).^11^, ^12^ Immune cell populations such as tumour‐associated macrophages and regulatory T cells (Tregs) can undergo functional reprogramming under specific metabolic states, thereby promoting tumour immune evasion and disease progression.^12^, ^13^, ^14^, ^15^ Particularly in HCC, an immunosuppressive microenvironment is considered one of the major factors limiting the efficacy of immunotherapy. Nevertheless, the key molecules linking tumour cell metabolic reprogramming to the establishment of an immunosuppressive TME in HCC, as well as their underlying mechanisms, remain to be further elucidated.

With the application of deep learning methods in biomedical research, nonlinear modelling of high‐dimensional omics data to extract latent features has become an important strategy for identifying key driver factors. Compared with conventional feature‐selection methods, deep autoencoders are better suited to capturing latent structural information embedded in complex expression profiles.^16^ Meanwhile, advances in single‐cell transcriptomics and spatial transcriptomics have provided new approaches for dissecting the state heterogeneity and spatial distribution of distinct cell populations within the tumour microenvironment.

To address these questions, we integrated metabolism‐related gene sets in HCC into functional metabolic modules and used an autoencoder to derive latent metabolic features from transcriptomic data. After prioritizing key metabolic programs with an XGBoost model, we identified TALDO1 as a key metabolism‐related gene in HCC. TALDO1 encodes transaldolase, a key enzyme in the nonoxidative branch of the pentose phosphate pathway that catalyses the reversible transfer of carbon skeletons among sugar phosphate intermediates.^17^, ^18^ By influencing the distribution of carbon flux among nucleotide biosynthesis, and the recycling of glycolytic intermediates, TALDO1 may support metabolic adaptation in tumour cells.^18^, ^19^, ^20^ Previous studies in HER2 positive breast cancer have shown that TALDO1 loss enhances oxidative stress and is accompanied by impaired lipid and nucleotide synthesis.^20^ In HCC, high TALDO1 expression is associated with poor prognosis and promotes tumour cell proliferation and migration.^21^, ^22^ However, the role of TALDO1 in metabolic reprogramming in HCC remains unclear. We therefore investigated the effects of TALDO1 on HCC metabolism. Our results showed that TALDO1 promoted lipogenesis and lipid accumulation in HCC cells by suppressing AMPK activation and maintaining SREBP1 maturation. TALDO1 silencing also altered the profile of lipids secreted by HCC cells, suggesting that lipid metabolic reprogramming driven by TALDO1 may be involved in remodelling the TME. On this basis, we performed single‐cell RNA sequencing on HCC specimens from our patient cohort and integrated spatial transcriptomic analysis to systematically characterize the association of TALDO1 with metabolic reprogramming and immune microenvironment remodelling. Multi‐omics analyses combined with multiplex immunofluorescence validation showed that high TALDO1 expression was closely associated with an immunosuppressive microenvironment in HCC. Further coculture experiments and orthotopic HCC models showed that TALDO1 silencing impaired the ability of HCC cells to induce M2‐like macrophage features and promote exhaustion‐associated phenotypes in CD8^+^ T cells, accompanied by enhanced local antitumour immune activity. Patient‐derived xenograft models further supported the tumour‐promoting role of TALDO1 in HCC.

Collectively, this study demonstrates that TALDO1 not only promotes lipid metabolic reprogramming in HCC, but is also closely associated with the establishment of an immunosuppressive TME. These findings suggest that TALDO1 may represent a key molecule linking metabolic abnormalities to microenvironmental remodelling and may have potential therapeutic value.

## 2 METHODS

### 2.1 Clinical specimens

Clinical specimens, including paired tumour tissues and adjacent non‐tumorous tissues, were collected from patients with HCC who underwent hepatectomy at the Second Qilu Hospital of Shandong University. Written informed consent was obtained from all patients. This study was conducted in accordance with the Declaration of Helsinki and was approved by the Scientific Research Ethics Committee of the Second Qilu Hospital of Shandong University (Approval No. KYLL202509708). After collection, part of each specimen was immediately snap‐frozen in liquid nitrogen for molecular analyses, whereas tissues used for histological and immunofluorescence staining were formalin‐fixed and paraffin‐embedded.

### 2.2 Tissue dissociation and single‐cell isolation

Fresh HCC specimens were obtained from four patients undergoing hepatectomy and subjected to single‐cell RNA sequencing. Tissue specimens were immediately preserved at low temperature after surgical excision and processed as soon as possible for single‐cell suspension preparation. Briefly, tissues were washed with pre‐chilled 1× DPBS, minced into small fragments, and incubated in freshly prepared digestion buffer with shaking at 37°C. The resulting cell suspension was filtered through a 70‐µm cell strainer, and cells were collected by centrifugation. Red blood cells were removed using red blood cell lysis buffer, followed by washing and resuspension in 1× DPBS containing 2% FBS. The resulting single‐cell suspension was subsequently used for library preparation.

### 2.3 Single‐cell RNA library construction and sequencing

Single‐cell suspensions with qualified viability were adjusted to an appropriate concentration and subjected to single‐cell capture and library construction using the DNBelab C‐TaiM4 platform. Individual cells were co‐encapsulated with barcoded gel beads to generate GEMs, within which reverse transcription and molecular barcoding were performed. The emulsions were then broken, followed by magnetic bead purification, recovery and amplification of first‐strand cDNA, and subsequent library construction after quality control. The final libraries were sequenced on the DNBSEQ platform in PE100 mode.

### 2.4 Single‐cell RNA sequencing data analysis

Raw sequencing data were initially processed using DNBc4tools for quality control, barcode identification, UMI quantification, and gene expression matrix construction, followed by alignment to the reference genome to generate single‐cell gene expression profiles. The resulting expression matrix was then analysed using Seurat v5 for quality control, normalization, dimensionality reduction and clustering. Cells with fewer than 500 detected genes, fewer than 1000 total RNA counts, or more than 10% mitochondrial gene expression were excluded from downstream analyses. Batch effects across samples were corrected using the Harmony algorithm. Clustering, UMAP visualization and downstream analyses were subsequently performed based on the Harmony corrected low dimensional embeddings. Marker genes for each cell cluster were identified using FindMarkers, and cell type annotation was performed based on canonical marker genes. Pseudotime trajectory analysis was conducted using Slingshot and Monocle2. For spatial transcriptomic data, spot‐level spatial coordinates, normalized expression matrices, and cell type annotations were extracted from the Seurat object. Spots annotated as differentiated malignant cells were defined as tumour spots. Spatial adjacency was established using a k‐nearest neighbour approach based on spot coordinates, and the median nearest‐neighbour distance across all spots was defined as the reference spot spacing. Two spots were considered directly adjacent when the distance between them did not exceed 1.15 times the reference spot spacing. Tumour spots directly adjacent to at least one non‐tumour spot were defined as tumour boundary spots. Among spots annotated as tumour‐associated macrophages (TAMs), the median TALDO1 expression level was calculated. TAM spots with TALDO1 expression above the median were classified as the TALDO1‐high TAM group, whereas the remaining TAM spots were assigned to the TALDO1‐low TAM group. For each TAM spot, the Euclidean distance to the nearest tumour boundary spot was calculated and normalized to the reference spot spacing. Differences in the distance to the tumour boundary between the two TAM groups were assessed using a two‐sided Wilcoxon rank‐sum test and further validated by 10 000 label permutations.

### 2.5 In silico perturbation analysis based on Geneformer

The raw count matrices, metadata and precomputed UMAP coordinates of myeloid cell subsets were exported for downstream analysis. To generate Geneformer input files, gene symbols were first converted to Ensembl IDs, and only genes supported by the Geneformer V2 tokenizer were retained together with cell barcode and cell type information to construct single‐cell input datasets. A total of 8417 myeloid cells were included in this analysis. The resulting datasets were then tokenized into rank‐value‐encoded expression profiles (maximum input length, 4096 tokens). For in silico perturbation, we used the publicly released, pretrained Geneformer V2 model (gf‐12L‐95 M‐i4096, a 12‐layer, 104‐million‐parameter transformer pretrained on approximately 95 million single‐cell transcriptomes) in zero‐shot mode, without any task‐specific fine‐tuning. To simulate TALDO1 knockout, the TALDO1 token was removed from the rank‐value‐encoded input sequence of each cell to generate a knockout‐tokenized dataset, and CLS embeddings were extracted using EmbExtractor (model_type = “Pretrained”, emb_mode = “cls”), producing one 512‐dimensional CLS embedding per cell for both the wild‐type and knockout states. For each target myeloid subset, the centroid was defined as the element‐wise arithmetic mean of the 512‐dimensional wild‐type CLS embeddings across all cells of that subset, and the cosine similarity between each cell's CLS embedding (E) and this centroid (C) was computed as $\cos(\theta )=\frac{E\cdot C}{∥E∥∥C∥}$. Directional shift was defined as the post‐knockout similarity to the centroid minus the pre‐knockout similarity, while shift magnitude was defined as its absolute value. Positive directional shifts indicate predicted movement toward the reference cell state and negative values movement away from it. The analysis was restricted to TALDO1‐positive cells, defined as cells with a raw TALDO1 UMI count ≥ 1. Per‐cell directional shifts were tested against zero using a two‐sided Wilcoxon signed‐rank test. *p* values across myeloid subsets were adjusted using the Benjamini‐Hochberg procedure, with an adjusted *p* value < .05 considered statistically significant.

### 2.6 Autoencoder model training and feature extraction

Previously reported metabolism‐related gene sets were integrated and classified into five metabolic modules according to their biological functions. RNA‐seq expression profiles and corresponding clinical information for the TCGA‐LIHC cohort were obtained from The Cancer Genome Atlas database. A total of 376 TCGA‐LIHC samples were included, and gene expression matrices corresponding to each metabolic module were extracted. Deep autoencoder models were subsequently applied to perform nonlinear dimensionality reduction of the high‐dimensional expression data, thereby generating low‐dimensional latent feature representations.

The model was designed with a symmetric encoder‐decoder architecture, and an independent feedforward neural network was constructed for each metabolic module. The encoder consisted of three hidden layers containing 32, 16 and 8 neurons, respectively. The samples were randomly divided into a training set and a validation set at a ratio of 8:2, comprising 300 and 76 samples, respectively. Model training was performed using the Adam optimizer with an initial learning rate of .001, a batch size of 64, and a maximum of 150 epochs. To reduce overfitting, a dropout rate of .1 was applied to each hidden layer. The model was optimized using the per‐feature reconstruction mean squared error as the loss function, and early stopping was implemented when no improvement in validation loss was observed for 10 consecutive epochs. The Captum framework was then used to quantify the contribution of each input gene to the latent features, thereby generating a gene importance matrix for each latent dimension. The extracted latent features were further subjected to univariate Cox regression analysis to evaluate their associations with overall survival, with statistical significance defined as *p* < .05. Prognosis‐related latent features identified from this analysis were subsequently incorporated into an XGBoost risk model to calculate risk scores for each patient. Patients were then stratified into high‐risk and low‐risk groups according to their risk scores for subsequent survival analysis and transcriptomic differential expression analysis.

### 2.7 Cell culture

The human hepatocellular carcinoma cell lines Huh7 and Hep3B, as well as the murine hepatoma cell line Hepa1‐6, were obtained from the Cell Bank of the Chinese Academy of Sciences. All cells were cultured under standard conditions in a humidified incubator at 37°C with 5% CO_2_ using DMEM medium (Gibco, Thermo Fisher Scientific) supplemented with 10% fetal bovine serum (FBS; Procell) and 1% penicillin‐streptomycin. All cell lines were authenticated by short tandem repeat profiling and were routinely tested for mycoplasma contamination to ensure accurate cell line identity and contamination‐free culture conditions. For pharmacological treatment, TALDO1 knockdown HCC cells were treated with the AMPK inhibitor Compound C (5 µM, 18 h; MCE, Cat. #HY‐13418A), with an equal volume of DMSO used as the vehicle control. Cells were collected after treatment, and the expression of proteins involved in AMPK/SREBP1 signalling and lipid synthesis was assessed by Western blotting.

### 2.8 Lentiviral transduction and establishment of stable cell lines

TALDO1 knockdown was induced in Huh7 and Hep3B cells by transduction with the corresponding lentiviral vectors (GenePharma). To improve transduction efficiency and enhance the reliability of the knockdown results, two independent shRNA constructs, shTALDO1‐1 and shTALDO1‐2, were used in parallel. For rescue experiments, stable TALDO1 knockdown cells were transduced with lentiviral vectors expressing TALDO1 or SREBP1 to generate TALDO1 rescue and SREBP1 rescue cell lines. At 48 h after lentiviral transduction, cells were selected with puromycin (4 µg/mL) for 7 days to establish stable cell lines. The shRNA sequences used in this study are listed in Table S1.

### 2.9 CRISPR/Cas9‐mediated TALDO1 knockout

sgRNAs targeting TALDO1 were designed and constructed according to the standard CRISPR/Cas9 cloning protocol established by the Feng Zhang laboratory. The sgRNA sequences (listed in Table S1) were cloned into the lentiCRISPR v2 vector (Addgene) for subsequent gene knockout experiments. Lentiviral particles were then packaged by polyethyleneimine (PEI)‐mediated transfection, and the viral supernatants were collected to infect target cells. The efficiency of TALDO1 knockout was verified by Western blot analysis.

### 2.10 RNA extraction and qRT‐PCR analysis

Total RNA was extracted from cells using TRIzol reagent (Vazyme) according to the manufacturer's instructions. RNA purity was assessed by measuring the absorbance ratio at 260/280 nm with a spectrophotometer, and RNA integrity was evaluated by agarose gel electrophoresis. After removal of genomic DNA, first‐strand cDNA was synthesized using HiScript II Q RT SuperMix (Vazyme). Quantitative real‐time PCR (qRT‐PCR) was then performed using ChamQ Universal SYBR qPCR Master Mix (Vazyme) on an ABI QuantStudio system or an equivalent platform. All reactions were performed in triplicate, with β‐actin used as the internal control. Relative gene expression levels were calculated using the 2^−ΔΔCt^ method. The primer sequences used for qRT‐PCR are listed in Table S2.

### 2.11 Western blot analysis

Cells were lysed using RIPA lysis buffer (Beyotime) supplemented with a protease inhibitor cocktail. After centrifugation at 12 000 × g for 10 min at 4°C, the supernatants were collected, and protein concentrations were determined using a BCA protein assay kit (Beyotime). Equal amounts of total protein (20–30 µg per lane) were separated by SDS‐PAGE and transferred onto PVDF membranes (Millipore). The membranes were blocked in TBST containing 5% (w/v) non‐fat milk for 1 h at room temperature and then incubated with primary antibodies overnight at 4°C (Table S3). After washing with TBST, the membranes were incubated with HRP‐conjugated secondary antibodies for 2 h at room temperature. Protein signals were detected using an enhanced chemiluminescence reagent (Vazyme), and digital images were acquired. β‐actin was used as the internal loading control.

### 2.12 Lipid staining and quantification

Intracellular neutral lipid droplets were stained with the lipophilic fluorescent probe BODIPY 493/503 (catalogue no. #C2053S, Beyotime) according to the manufacturer's instructions, and images were acquired using a fluorescence microscope. Intracellular triglyceride (TG), polyunsaturated fatty acid (PUFA), and free fatty acid levels were quantitatively measured using the corresponding commercial assay kits according to the manufacturers’ instructions (catalogue nos. #A110‐1‐1 and #A042‐2‐1; #CB17577‐Hu, CoiboBio).

### 2.13 Wide‐targeted lipidomics analysis

Control and TALDO1 knockdown Huh7 cells were seeded in culture dishes at the same initial cell density. After cell attachment and attainment of comparable confluence, the culture medium was replaced with serum‐free medium, and cells were cultured for an additional 24 h. Three independent biological replicates were included for each group, and conditioned media were collected under identical medium volumes and culture conditions. The collected conditioned media were centrifuged to remove suspended cells and cellular debris, after which the supernatants were subjected to lipid extraction. Lipids were extracted using a modified methyl tert‐butyl ether (MTBE)/methanol/water protocol. Following phase separation, the upper organic phase was collected, dried under nitrogen, and reconstituted in an appropriate solvent for analysis. Widely targeted lipidomics was performed using an ACQUITY UPLC I‐Class PLUS system (Waters, USA) coupled with a QTRAP 6500+ mass spectrometer (SCIEX) operated in scheduled multiple reaction monitoring mode. This approach enabled sensitive and high‐throughput quantification of a broad range of lipid species across multiple lipid classes.

### 2.14 Immune cell coculture assays

For macrophage coculture experiments, THP‐1 cells were cultured in RPMI‐1640 medium supplemented with 10% fetal bovine serum and 55 µM β‐mercaptoethanol. To induce differentiation into unpolarized M0 macrophages, THP‐1 cells were seeded in 24‐well plates in 0.3 mL of medium per well and treated with phorbol 12‐myristate 13‐acetate (PMA; 50 ng/mL) for 48 h. A Transwell based coculture system was established using Huh7 cells and THP‐1‐derived macrophages. THP‐1‐derived M0 macrophages were maintained in the lower chamber of 24‐well Transwell plates, while control (NC) or TALDO1 knockdown Huh7 cells were seeded in the upper chamber at a 1:1 ratio. Cocultures were maintained for 48 h in RPMI‐1640 medium supplemented with 10% fetal bovine serum and 55 µM β‐mercaptoethanol. After coculture, only macrophages from the lower chamber were collected for subsequent analyses. Human CD8^+^ T cells were isolated from peripheral blood mononuclear cells using an immunomagnetic separation kit (Human CD8^+^ T Cells Negative Selection Kit, MCE, HY‐K0351). Peripheral blood mononuclear cells were resuspended in sorting buffer and incubated with a biotinylated antibody cocktail at room temperature for 15 min. After centrifugation and removal of the supernatant, the cell pellet was resuspended in fresh sorting buffer. Streptavidin‐conjugated magnetic beads were then added, followed by a further 15‐min incubation at room temperature. The tube was subsequently placed on a magnetic stand for 5 min. Bead‐bound non‐CD8^+^ cells were retained on the tube wall, whereas the unbound cells in the supernatant were collected as enriched CD8^+^ T cells. The isolated cells were finally resuspended in 1 mL of complete medium. Meanwhile, CD8^+^ T cells were activated using anti‐CD3/CD28 antibodies and recombinant human IL‐2. A similar Transwell based coculture system was established using CD8^+^ T cells and NC or TALDO1 knockdown Huh7 cells.

### 2.15 In vivo experiments

All animal experiments were approved by the Animal Ethics Committee of the Second Qilu Hospital of Shandong University. Six‐week‐old C57BL/6 mice were used in this study. Hepa1‐6 cells used for in vivo experiments included WT and TALDO1‐KO cells. For the subcutaneous tumour model, WT or TALDO1‐KO Hepa1‐6 cells were resuspended in sterile PBS and subcutaneously injected into mice at a dose of 3 × 10^6^ cells in 200 µL PBS. Tumour growth was monitored every 3 days, and tumour length and width were measured using digital callipers. At the experimental endpoint, mice were sacrificed, and tumour tissues were excised and weighed. Part of the tumour tissues was used for histological staining analysis. For the experimental lung metastasis model, luciferase‐labelled WT or TALDO1‐KO Luc‐Hepa1‐6 cells were resuspended in sterile PBS and injected into C57BL/6 mice via the tail vein at a dose of 1 × 10^6^ cells in 150 µL PBS. Four weeks later, lung metastasis‐associated fluorescence signals were detected using a small‐animal in vivo imaging system, after which the mice were euthanized, and lung tissues were collected. For the orthotopic liver implantation model, WT or TALDO1‐KO Hepa1‐6 cells were injected into the livers of C57BL/6 mice to establish an orthotopic liver cancer model. After model establishment, orthotopic tumour growth was monitored every 3 days using a small‐animal in vivo imaging system. At the end of the experiment, mice were euthanized, and tumour tissues were collected for flow cytometry and multiplex immunofluorescence staining.

### 2.16 PDX model and recombinant AAV shRNA vector construction

To establish the PDX model, tumour fragments obtained from a patient with HCC were subcutaneously implanted into NCG mice. Adeno associated virus serotype 8 (AAV8) was used as the vector for in vivo experiments. An shRNA sequence targeting TALDO1 or a nontargeting control shRNA sequence was cloned into the AAV8 vector. The resulting constructs were transfected into HEK 293 cells to generate AAV shTALDO1 and AAV shNC preparations. The viral preparations were confirmed to be free of replication competent virus, endotoxin and bacterial contamination.

When PDX tumours reached approximately 60 to 120 mm^3^, mice were randomly assigned to two groups, with five mice per group. Mice then received intratumoral injections of AAV shTALDO1 or AAV shNC at a volume of 100 µL per injection and a titter of 4 × 10^1^ ^1^ viral particles/mL. Injections were administered every other day for a total of four doses. Tumour volumes were measured every 3 days. At the experimental endpoint, mice were euthanized, tumours were excised, and tumour weights were recorded.

### 2.17 Multiplex immunofluorescence staining

Human and murine HCC tissue specimens were formalin‐fixed, paraffin‐embedded, and cut into 4‐µm‐thick sections. After deparaffinization, rehydration, antigen retrieval and blocking, the tissue sections were stained using a multiplex immunofluorescence staining kit according to the manufacturer's instructions. The sections were then sequentially incubated with the indicated antibodies, followed by fluorescence signal development, and nuclei were counterstained with DAPI. After staining, images were acquired using a fluorescence microscope and further processed using image analysis software. M2 macrophages and exhausted T cells were identified based on the marker combinations CD68/CD206 and CD8/PD‐1, respectively. Image analysis was performed using ImageJ software. Colocalization of TALDO1 with CD68^+^CD206^+^ or CD8^+^PD‐1^+^ regions was assessed using the Coloc2 plugin and quantified using Manders’ coefficient. Five randomly selected fields were analysed for each tissue specimen, and their mean value was used as the representative value for that specimen. Differences in Manders’ coefficients and positive cell proportions between paired tissues were evaluated using a two‐sided paired Student's *t*‐test. Detailed information on the antibodies used and their working concentrations is provided in Table S3.

### 2.18 Flow cytometry analysis

Orthotopic liver tumour tissues and immune cells collected from coculture experiments were subjected to flow cytometric analysis. Orthotopic liver tumours were mechanically dissociated to generate single cell suspensions, which were passed through cell strainers to remove tissue debris and cell aggregates, followed by red blood cell lysis. After washing, cells were resuspended in staining buffer and incubated with the appropriate fluorochrome conjugated antibodies for surface marker staining. For intracellular marker detection, cells were fixed, permeabilized and stained according to the manufacturer's instructions after surface staining. For coculture experiments, THP‐1‐derived macrophage‐like cells and human CD8^+^ T cells were collected separately from the lower chamber of the Transwell system. Cell debris, doublets and nonviable cells were excluded before subsequent staining and analysis. Following staining, samples were washed and acquired on a flow cytometer. All flow cytometry data were analysed using FlowJo software (version 10.7.1).

According to the predefined gating strategy, Treg cells were defined as CD25^+^FOXP3^+^ cells within the CD45^+^CD3^+^CD4^+^ population. CD8^+^IFN‐γ^+^ and CD8^+^GZMB^+^ T cells were assessed within the CD45^+^CD3^+^ population. M2‐like and M1‐like macrophages were defined as CD206^+^ and CD86^+^ cells, respectively, within the CD45^+^CD11b^+^F4/80^+^ population. Activated dendritic cells were defined as CD80^+^CD86^+^ cells within the CD45^+^CD11c^+^MHC‐II^+^ population. Details of the antibodies used are provided in Table S3.

### 2.19 RNA sequencing and quantitative analysis

Total RNA was extracted from Huh7 cells in the TALDO1 knockdown group and the control group using TRIzol reagent (Invitrogen), with three biological replicates included in each group. After assessment of RNA purity and integrity, mRNA was enriched using oligo(dT) magnetic beads and subsequently fragmented. Sequencing libraries were then constructed, purified with magnetic beads, and subjected to paired‐end 150 bp (PE150) sequencing on the Illumina NovaSeq 6000 platform. After quality control filtering, the raw sequencing reads were aligned to the human reference genome (hg38) using HISAT2 (v2.2.0). The alignment files were sorted with Samtools (v1.9) and visualized using IGV. Transcript assembly was performed with StringTie (v1.3.0), and differential expression analysis was conducted using edgeR (v3.36.0). Genes with an adjusted *p* value < .05 and a fold change > 2 were defined as differentially expressed genes.

### 2.20 Immunohistochemical staining

Paraffin‐embedded tissue samples fixed in formalin were cut into 4‐µm sections and mounted onto glass slides. The sections were deparaffinized in xylene, rehydrated through graded ethanol, and subjected to heat‐mediated antigen retrieval in citrate buffer. To eliminate endogenous peroxidase activity, the slides were treated with 3% hydrogen peroxide. After incubation with primary antibodies, HRP‐conjugated secondary antibodies were applied using the UltraSensitive™ S‐P kit (Beyotime). Immunostaining was visualized with DAB, followed by haematoxylin counterstaining of nuclei. The stained sections were then dehydrated, mounted, and imaged.

### 2.21 Statistical analysis

Statistical analyses were performed using GraphPad Prism 8 and R software (version 4.1.1). Comparisons between two groups were conducted using a two‐sided Student's *t*‐test, whereas comparisons among multiple groups were performed using one‐way analysis of variance. Tumour growth curves were analysed using two‐way repeated‐measures analysis of variance. Correlations between variables were assessed using Spearman correlation analysis. Data from in vitro experiments are presented as the mean ± standard deviation from at least three independent biological replicates. A *p* value ≤ .05 was considered statistically significant.

## 3 RESULTS

### 3.1 Deep learning‐based identification of metabolism‐associated features

**FIGURE 1:**
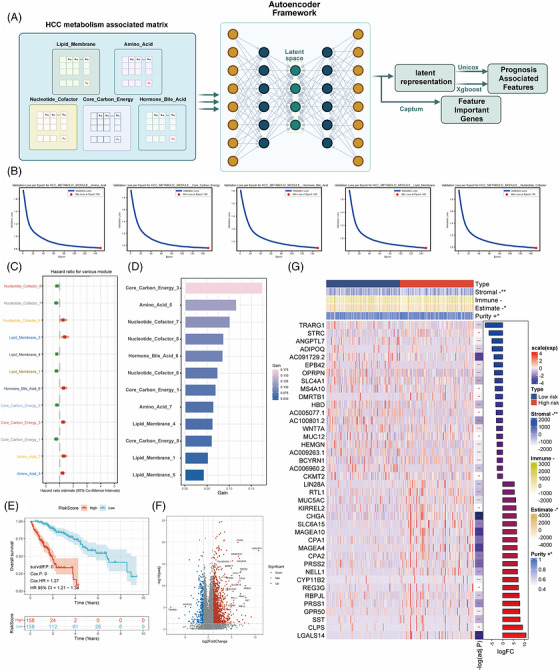
Deep learning–based identification of metabolism‐associated features. (A) Analytical framework integrating metabolism‐related functional modules and performing nonlinear dimensionality reduction using a deep autoencoder model. (B) Loss function curve during model training. (C) Univariate Cox regression analysis of the latent features obtained after dimensionality reduction. (D) Feature importance ranking in the XGBoost model. (E) Overall survival curves of the high‐risk and low‐risk groups. (F) Volcano plot of differentially expressed genes between the high‐risk and low‐risk groups. (G) Comparison of tumour microenvironment–related scores and tumour purity between the high‐ and low‐risk groups. *p < 0.05, **p < 0.01, and ***p < 0.001.

Given the prominent metabolic reprogramming characteristics of HCC, we first integrated previously reported metabolism‐related gene sets and classified them into five metabolic functional modules according to their biological functions (Figure 1A, Table S4). Based on transcriptomic sequencing datasets of HCC, expression matrices of genes within these metabolic modules were constructed. To capture the latent structural features embedded in the high‐dimensional expression matrices, we developed a deep autoencoder model to perform nonlinear dimensionality reduction on the expression profiles of different metabolic modules, thereby generating low‐dimensional latent representations (Figure 1A). To enhance model interpretability, feature attribution analysis was conducted using the Captum framework to derive gene importance rankings corresponding to the latent representations. During model training, the loss function continuously decreased and gradually stabilized across iterations, indicating favourable convergence and generalization performance of the model (Figure 1B).

Univariate Cox regression analysis of the derived latent features revealed that Core_Carbon_Energy_3, Lipid_Membrane_5, and Hormone_Bile_Acid_6 were significantly associated with unfavourable prognosis in patients with HCC (Figure 1C). Furthermore, an XGBoost‐based risk model was constructed using the prognostically relevant features, in which Core_Carbon_Energy_3 exhibited the highest contribution to the model, suggesting a potentially central role in HCC progression (Figure 1D, Figure S1A). Based on the calculated risk scores, patients were stratified into high‐risk and low‐risk groups. Kaplan–Meier survival analysis demonstrated that patients in the high‐risk group had significantly poorer overall survival compared with those in the low‐risk group (Figure 1E). Differential expression analysis further indicated substantial transcriptomic differences between the two risk groups (Figure 1F).

To further elucidate the biological distinctions between the high‐ and low‐risk groups, we systematically evaluated tumour microenvironment‐related characteristics. The results showed that the high‐risk group exhibited significantly reduced immune‐ and stromal‐related scores, accompanied by a marked increase in tumour purity, suggesting that high‐risk tumours may display an immunosuppressive, stroma‐depleted phenotype with a higher proportion of malignant cells (Figure 1G).

### 3.2 TALDO1 is a key metabolism‐related gene that drives hepatocellular carcinoma progression

**FIGURE 2:**
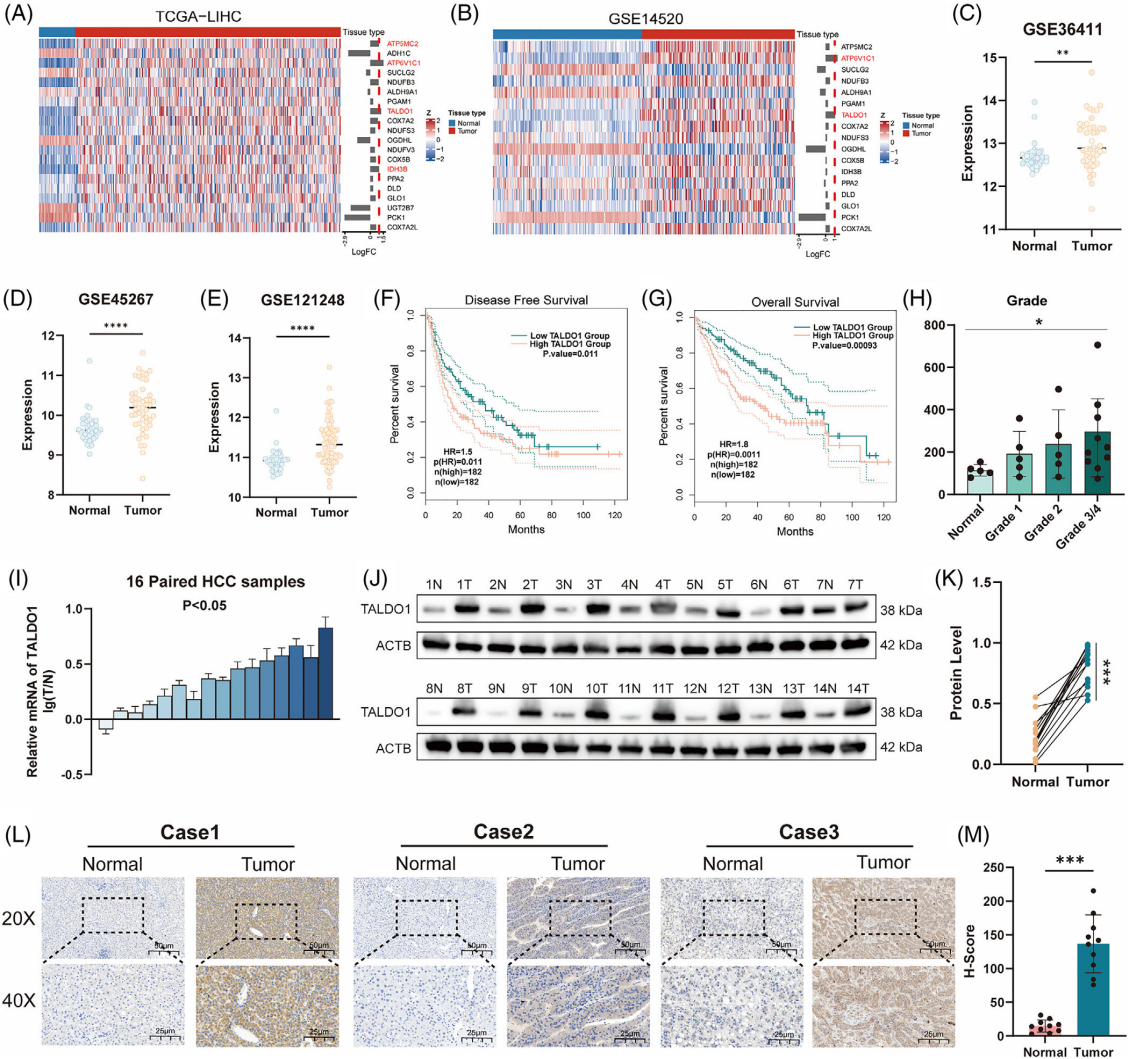
TALDO1 is identified as a key energy metabolism–associated gene in hepatocellular carcinoma. (A and B) Expression differences of the top 20 candidate genes from Core_Carbon_Energy_3 between hepatocellular carcinoma and normal tissues in the TCGA‐LIHC and GSE14520 cohorts. (C–E) Validation of TALDO1 expression across three independent transcriptomic datasets. (F–G) Kaplan–Meier analysis of disease‐free survival (DFS) and overall survival (OS) according to TALDO1 expression levels. (H) TALDO1 expression was significantly positively correlated with tumour grade. (I) Differences in TALDO1 transcript levels in paired clinical samples (*n* = 16 per group). (J and K) Differences in TALDO1 protein expression levels in paired clinical samples (*n* = 14 per group). (L) Representative images of TALDO1 immunohistochemical staining in clinical samples. (M) Quantification of TALDO1 immunohistochemical staining (*n* = 10 per group). *p < 0.05, **p < 0.01, and ***p < 0.001.

Given that Core_Carbon_Energy_3 was identified as a significant prognostic risk feature and exhibited the highest contribution in the XGBoost model, we further focused on this metabolic program to screen for its potential key driver genes. Based on the feature importance ranking derived from the deep learning model, the top 20 genes were selected for subsequent analyses (Table S5). Analysis of two independent cohorts, TCGA‐LIHC and GSE14520, revealed that only ATP6V1C1 and TALDO1 consistently showed elevated expression in hepatocellular carcinoma tissues (Figure 2A,B). Kaplan–Meier survival analysis further demonstrated that high TALDO1 expression was significantly associated with unfavourable DFS and OS (Figure 2F,G), whereas ATP6V1C1 expression showed no significant association with survival outcomes (Figure S1B,C). In addition, its elevated expression in tumour tissues was further validated across three independent transcriptomic datasets (GSE45267, GSE36411 and GSE121248) (Figure 2C–E). Meanwhile, TALDO1 expression increased significantly with increasing tumour grade (Figure 2H). Notably, TALDO1 expression was significantly higher in both HBV related and NAFLD related HCC than in normal liver tissues, suggesting that its aberrant upregulation may represent a common feature of HCC across distinct etiological backgrounds (Figure S2A).

To further confirm the expression pattern of TALDO1 in clinical specimens, we analysed 16 pairs of hepatocellular carcinoma tissues and matched adjacent non‐tumorous liver tissues obtained from the biobank of Qilu Second Hospital of Shandong University. qRT‐PCR analysis showed that TALDO1 transcript levels were significantly increased in tumour tissues compared with adjacent tissues (Figure 2I). Consistently, western blot analysis further confirmed a marked upregulation of TALDO1 protein levels in 14 paired samples (Figure 2J,K). In addition, immunohistochemical (IHC) staining of three representative paired specimens further validated the elevated expression of TALDO1 in tumour tissues (Figure 2L,M). Collectively, these results suggest that TALDO1 may represent a key energy metabolism‐associated gene involved in hepatocellular carcinoma progression.

### 3.3 TALDO1 promotes lipid metabolic reprogramming in HCC cells

**FIGURE 3:**
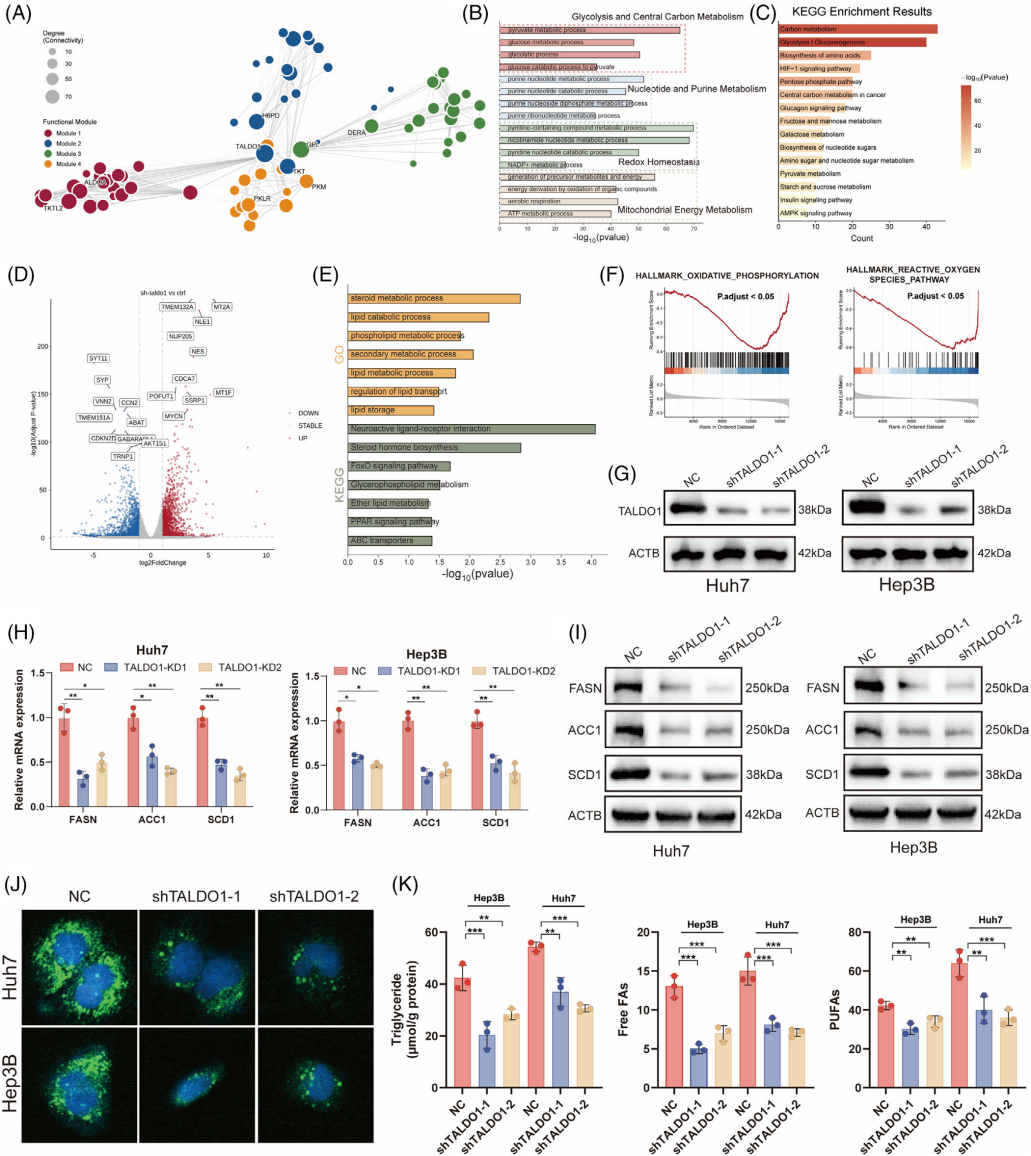
TALDO1 promotes lipid metabolic reprogramming in HCC. (A) PPI network of TALDO1. (B) GO enrichment analysis of TALDO1 and its interacting genes. (C) KEGG pathway enrichment analysis of TALDO1 and its interacting genes. (D) Volcano plot of transcriptome changes in TALDO1‐KD versus TALDO1‐WT Huh7 cells. (E) GO and KEGG enrichment analysis of downregulated genes in TALDO1‐KD cells. (F) GSEA of DepMap CRISPR knockout data for TALDO1. (G) Western blot validation of TALDO1 knockdown in Hep3B and Huh7 cells. (H) qPCR analysis of FASN, ACC1, and SCD1 mRNA expression in HCC cells following TALDO1 knockdown. (I) WB analysis showing decreased expression of lipogenic enzymes FASN, ACC1, and SCD1 in TALDO1‐KD cells. (J) BODIPY staining showing reduced lipid accumulation in TALDO1‐KD cells. (K) Reduced TG, Free FAs, and PUFAs in TALDO1‐KD cells. *p < 0.05, **p < 0.01, and ***p < 0.001.

To investigate the oncogenic role of TALDO1 in HCC, we first constructed a PPI network for TALDO1 (Figure 3A). GO and KEGG enrichment analyses of TALDO1 and its interacting genes revealed significant activation of carbon metabolism, mitochondrial energy metabolism and related metabolic pathways (Figure 3B–C), suggesting a potential role for TALDO1 in metabolic reprogramming. We then performed transcriptome sequencing of TALDO1‐knockdown (TALDO1‐KD) and TALDO1‐WT Huh7 cells. Volcano plot analysis identified extensive gene downregulation (Figure 3D). GO and KEGG enrichment of the downregulated genes showed significant enrichment of lipid metabolism and transport, lipid storage and PPAR signalling pathways (Figure 3E), indicating that TALDO1 may promote lipid metabolic reprogramming in HCC cells through regulation of lipid metabolism‐related genes.

In addition, analysis of DepMap CRISPR knockout data revealed that cells with high TALDO1 expression exhibited increased dependency on genes involved in oxidative phosphorylation and ROS response (Figure 3F), suggesting that TALDO1 may be associated with cellular energy stress regulation. To experimentally validate the role of TALDO1 in HCC lipid metabolism, we established stable TALDO1‐KD HCC cell lines (Figure 3G). qRT‐PCR analysis showed that TALDO1 knockdown significantly reduced the mRNA levels of FASN, ACC1, and SCD1 in both Huh7 and Hep3B cells (Figure 3H), indicating suppression of the lipogenic transcriptional program. Consistently, WB analysis showed reduced protein expression of FASN, ACC1, and SCD1 following TALDO1 knockdown (Figure 3I). BODIPY staining further demonstrated a marked decrease in intracellular lipid accumulation in TALDO1 knockdown cells (Figure 3J). Quantitative lipid analysis confirmed that intracellular triglyceride, free fatty acid and polyunsaturated fatty acid levels were all reduced after TALDO1 knockdown (Figure 3K).

Collectively, TALDO1 depletion suppressed the expression of lipogenesis related genes and key enzymes and reduced intracellular lipid accumulation in HCC cells, suggesting that TALDO1 may contribute to lipid metabolic reprogramming in HCC.

### 3.4 TALDO1 regulates lipid metabolic reprogramming in HCC through AMPK/SREBP1 Signalling

**FIGURE 4:**
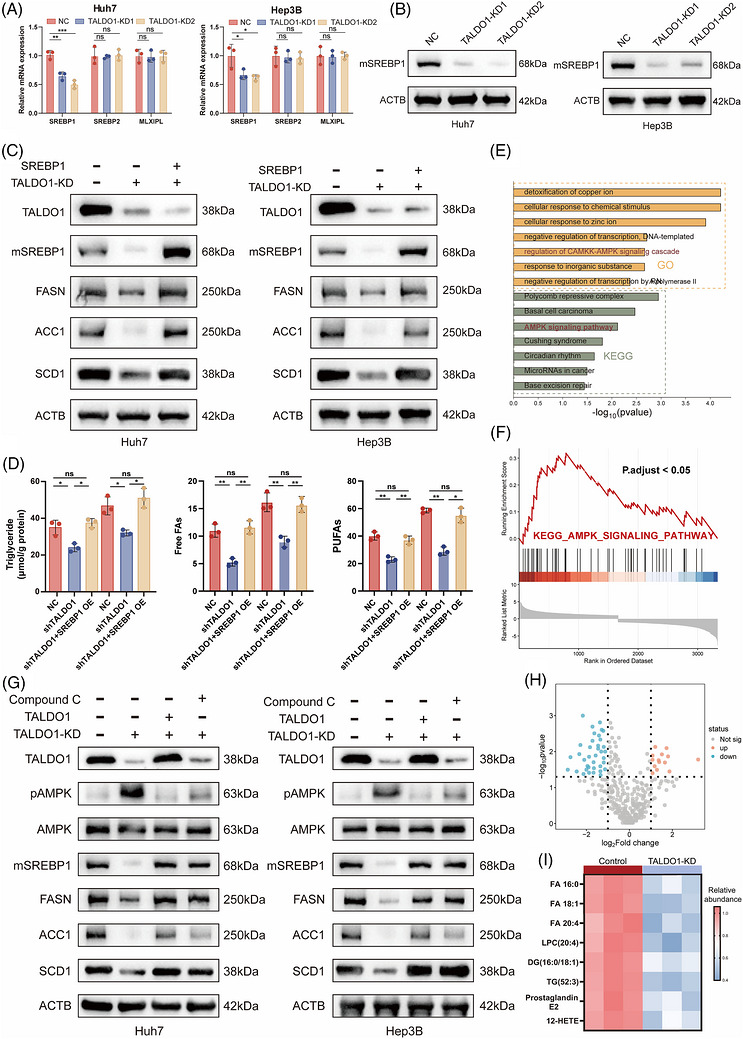
TALDO1 regulates AMPK/SREBP1 signalling‐mediated lipogenesis. (A) qRT‐PCR analysis of SREBP1, SREBP2 and MLXIPL mRNA expression in HCC cells following TALDO1 knockdown. (B) Protein expression of mSREBP1 following TALDO1 knockdown. (C) Effects of SREBP1 re‐expression on the expression of lipogenic enzymes in TALDO1‐KD cells. (D) Effects of SREBP1 re‐expression on triglyceride, free fatty acid and polyunsaturated fatty acid levels in TALDO1‐KD cells. (E) GO and KEGG enrichment analyses of genes upregulated following TALDO1 knockdown. (F) GSEA of the AMPK signalling pathway in TALDO1‐knockdown cells. (G) TALDO1 knockdown activated AMPK and suppressed mSREBP1 and downstream lipogenic enzyme expression, whereas TALDO1 re‐expression or AMPK inhibition partially reversed these changes. (H and I) Volcano plot and heatmap showing differential lipids in conditioned media from control and TALDO1‐KD HCC cells. *p < 0.05, **p < 0.01, and ***p < 0.001.

Given that TALDO1 functions in the nonoxidative branch of the pentose phosphate pathway, we first assessed whether TALDO1 mediated regulation of lipid metabolism in HCC is primarily associated with NADPH homeostasis or insufficient carbon precursors. In HCC cells, the decrease in the NADPH/NADP^+^ ratio following TALDO1 knockdown did not reach statistical significance (Figure S2B). In addition, supplementation with acetate or citrate did not significantly restore the reduced lipid metabolic phenotypes in TALDO1 knockdown cells (Figure S2C). Consistently, qPCR results showed that acetate or citrate did not significantly restore the mRNA expression of lipogenic enzymes (Figure S2D). These findings suggest that the regulatory effects of TALDO1 on lipid metabolism in HCC cannot be attributed solely to altered NADPH/NADP^+^ homeostasis or insufficient substrates for fatty acid synthesis. We therefore further investigated the effects of TALDO1 on lipogenic transcription factors to elucidate the potential molecular mechanism underlying TALDO1 regulation of lipid metabolism. qPCR showed that TALDO1 knockdown significantly reduced SREBP1 mRNA levels in HCC cells, whereas SREBP2 and MLXIPL did not show consistent changes (Figure 4A). WB further demonstrated that TALDO1 knockdown markedly decreased mature SREBP1 (mSREBP1) protein levels (Figure 4B). To determine whether SREBP1 contributes to TALDO1‐mediated lipogenesis, SREBP1 was re‐expressed in cells with TALDO1 knockdown. SREBP1 overexpression restored the protein levels of lipogenic genes (Figure 4C) and significantly attenuated the reductions in triglycerides, free fatty acids and polyunsaturated fatty acids induced by TALDO1 knockdown (Figure 4D), indicating that SREBP1 acts downstream of TALDO1 in the regulation of lipogenesis.

To investigate the upstream mechanism through which TALDO1 regulates SREBP1, we performed functional enrichment analysis of genes upregulated following TALDO1 knockdown. GO and KEGG analyses showed significant enrichment of the AMPK signalling pathway (Figure 4E). GSEA likewise showed positive enrichment of the AMPK signalling pathway in TALDO1 knockdown cells (Figure 4F). Given the role of AMPK in energy sensing and lipogenic transcriptional control, we next examined AMPK signalling.^23^ WB showed that TALDO1 knockdown increased p‐AMPK levels, accompanied by reduced expression of mSREBP1, FASN, ACC1 and SCD1 (Figure 4G). Re‐expression of TALDO1 reversed these changes (Figure 4G). In addition, treatment with the AMPK inhibitor Compound C partially reduced p‐AMPK levels and restored the expression of mSREBP1 and lipogenic enzymes (Figure 4G). Consistently, Compound C treatment partially restored intracellular lipid levels in HCC cells with TALDO1 knockdown (Figure S3A). In addition, Compound C treatment increased the mRNA expression levels of FASN, ACC1, and SCD1 in TALDO1 silenced HCC cells (Figure S3B,C). These findings suggest that AMPK activation induced by TALDO1 loss may contribute to the suppression of mSREBP1 and its downstream lipogenic program, supporting a role for the AMPK/SREBP1 axis in TALDO1‐mediated lipid metabolic reprogramming in HCC.

In addition, lipidomic analysis was performed using conditioned media collected from control and TALDO1 knockdown Huh7 cells. The volcano plot revealed significant changes in the abundance of multiple lipid species following TALDO1 knockdown (Figure 4H). The heatmap showed an overall decrease in fatty acids, triglycerides, and lipid mediators such as PGE2 (Figure 4I). These findings suggest that TALDO1 loss remodels the secreted lipid profile of HCC cells and may be associated with altered lipid signalling in the TME.

### 3.5 Expression and regulatory role of TALDO1 in the tumour microenvironment of HCC

**FIGURE 5:**
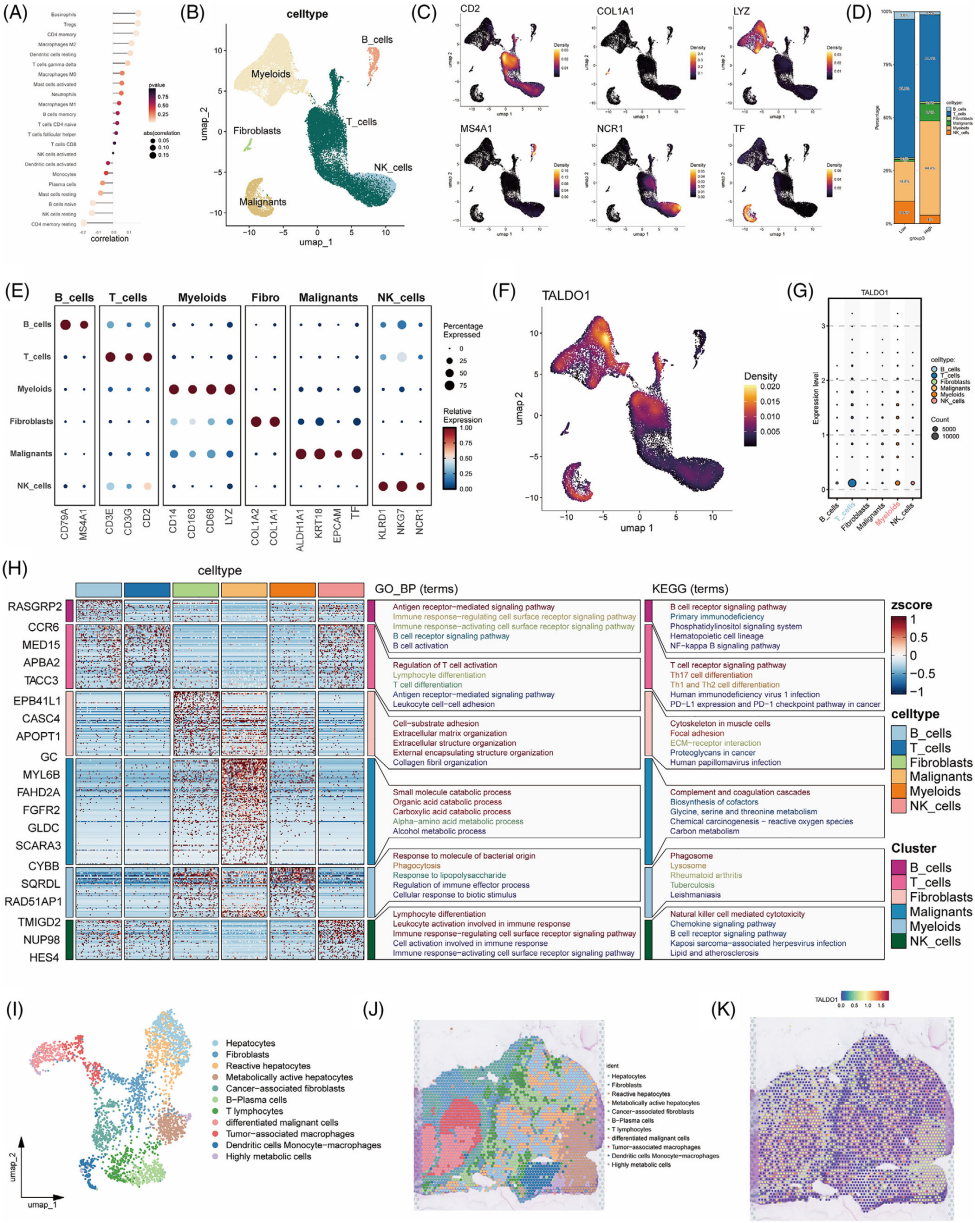
Expression and distribution of TALDO1 in the tumour microenvironment of HCC. (A) Correlation analysis between TALDO1 expression and tumour immune cell infiltration. (B and C) Cell type annotation and distribution based on scRNA‐seq data. (D) Proportions of different cell types in TALDO1 high‐ and low‐expression groups. (E) Bubble plot of marker genes for major cell types. (F and G) Expression distribution of TALDO1 across different cell types. (H) Heatmap of functional feature differences among different cell types. (I and J) Identification and spatial distribution of cell types in spatial transcriptomic data. (K) Expression characteristics of TALDO1 across different spatial cell types.

Given that TALDO1 is involved in metabolic regulation and metabolic reprogramming is considered an important factor in reshaping the tumour microenvironment, we therefore investigated the relationship between TALDO1 expression and tumour immune infiltration.^24^ The results showed that TALDO1 expression was correlated with the infiltration of multiple immune cell types, suggesting that TALDO1 may be associated with immune microenvironment remodelling (Figure 5A). Spearman correlation analysis showed that TALDO1 expression was positively correlated with several immune checkpoint molecules, including CTLA4, HAVCR2 and TIGIT (Figure S5C). These findings suggest that tumours with high TALDO1 expression may be associated with immune states relevant to the response to immune checkpoint blockade.

To further investigate the impact of TALDO1 on the tumour microenvironment, we performed single‐cell RNA sequencing on HCC specimens obtained from four patients who underwent curative hepatectomy and integrated these data with the publicly available dataset GSM5076749. UMAP plots before and after Harmony correction showed reduced separation between cells from the in‐house samples and public datasets, improved mixing of shared cell populations, and preservation of the overall cell type structure (Figure S3D–E). Based on marker genes, cells were classified into six major cell types (Figure 5B,C). A marker gene bubble plot confirmed the accuracy of the cell‐type annotations (Figure 5E). In addition, all cells were stratified into TALDO1‐high and TALDO1‐low groups, revealing an increased proportion of malignant cells and decreased proportions of T cells and NK cells in the TALDO1‐high group (Figure 5D), indicating an association between TALDO1 and tumour microenvironment remodelling. Meanwhile, TALDO1 was highly expressed in myeloid cells and T cells (Figure 5F,G). A heatmap illustrated functional differences among the different cell types (Figure 5H).

Subsequently, we analysed the spatial transcriptomics dataset GSE224411 to characterize the spatial expression pattern of TALDO1. Unsupervised clustering of the ST data identified a total of 11 spatial subclusters (Figure S4A). Based on specific marker genes, 11 major cell types were further annotated (Figure 5I,J, Figure S4B). The results showed that TALDO1 was significantly enriched in tumour‐associated macrophages (Figure 5K). Boundary proximity analysis further showed that TALDO1‐high tumour‐associated macrophages were preferentially located near tumour boundaries (Figure S4C), suggesting that they may contribute to shaping the microenvironment at the tumour border. Collectively, these findings indicate that TALDO1 is potentially associated with an immunosuppressive tumour microenvironment and may influence tumour microenvironment remodelling.

### 3.6 TALDO1 is associated with macrophage mediated immunosuppressive remodelling

**FIGURE 6:**
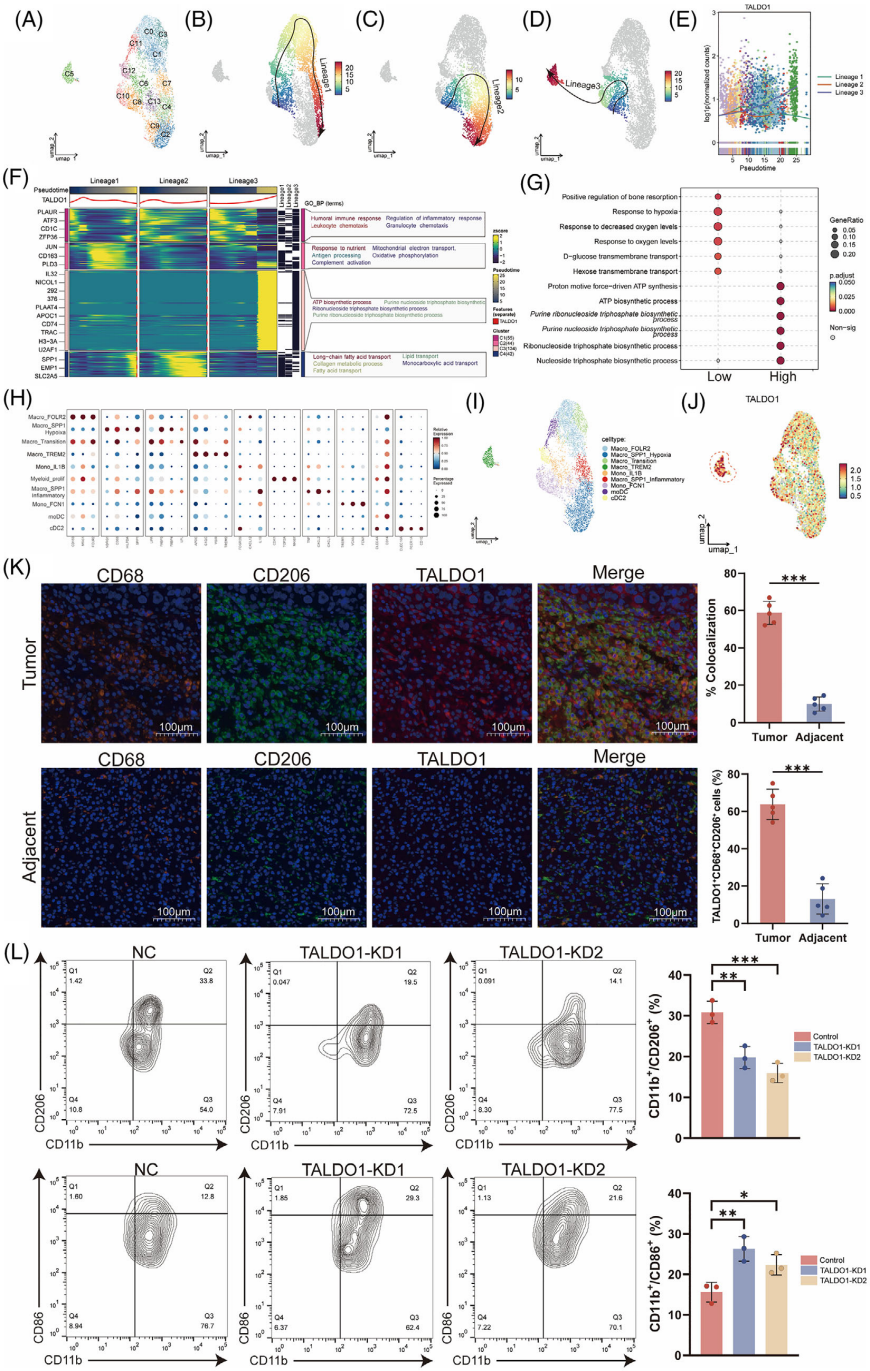
TALDO1 characterizes an immunosuppressive macrophage‐associated state in HCC. (A) Subclustering of myeloid cells. (B–D) Pseudotime differentiation trajectory analysis of myeloid cells. (E) Dynamic expression changes of TALDO1 along different pseudotime trajectories. (F) Pathway enrichment analysis of different pseudotime trajectories. (G) Differences in pathway activities between TALDO1 high and low expression myeloid cells. (H) Bubble plot of marker genes for major myeloid cell types. (I) UMAP showing annotated myeloid cell types. (J) TALDO1 expression in different myeloid subsets. (K) Representative mIF images of CD68, CD206 and TALDO1 staining in HCC tumour tissues and paired adjacent tissues. The right panels show quantitative analyses of the colocalization between TALDO1 and CD68^+^CD206^+^ cells, as well as the proportion of TALDO1^+^CD68^+^CD206^+^ cells. For each sample, measurements from five randomly selected fields were averaged for statistical analysis (*n* = 5 per group). (L) PMA‐induced THP‐1‐derived macrophage‐like cells were co‐cultured with control or TALDO1‐silenced Huh7 cells in a Transwell system. CD11b, CD206 and CD86 expression was assessed by flow cytometry (*n* = 3 per group). *p < 0.05, **p < 0.01, and ***p < 0.001.

Given the high expression of TALDO1 in myeloid cells and the recognized role of metabolic reprogramming in macrophage‐mediated immune regulation, we further investigated the expression pattern and potential function of TALDO1 in myeloid cells. Myeloid cells were subdivided into 14 subclusters (Figure 6A), and three pseudotime differentiation trajectories were constructed (Figure 6B–D). The results showed that TALDO1 expression continuously increased along the differentiation trajectory in Lineage 3 (Figure 6E). Pathway enrichment analysis across different pseudotime trajectories revealed that, compared with the other lineages, pathways related to energy metabolism and nucleotide biosynthesis were significantly activated in Lineage 3 (Figure 6F), suggesting that TALDO1 may be closely associated with a myeloid cell functional state characterized by specific metabolic features.

In addition, myeloid cells were stratified according to TALDO1 expression levels. The results showed that metabolism‐related pathways associated with immunosuppression were significantly enriched in the TALDO1‐high group (Figure 6G). Based on specific marker genes, myeloid cells were further classified into nine major cell types (Figure 6H,I). Notably, TALDO1 exhibited significantly higher expression in TREM2^+^ macrophages (Figure 6J). Furthermore, in the GSE149614 dataset, we identified a TALDO1^+^TREM2^+^ macrophage subset characterized by the expression of canonical marker genes (Figure S4D–F). This subset was significantly more abundant in HCC tissues than in normal liver tissues (Figure S4G), further supporting its reproducible presence across independent cohorts. Given that TREM2^+^ macrophages are widely recognized as possessing an M2‐like immunosuppressive phenotype, these findings suggest that TALDO1 may be associated with an immunosuppressive macrophage state.

To further explore the potential impact of TALDO1 perturbation on myeloid cell state representations, we performed Geneformer based in silico token perturbation in all myeloid cells. The analysis predicted heterogeneous cell state responses after TALDO1 token deletion. Specifically, TREM2^+^ macrophages showed a predicted negative shift, whereas most other myeloid cell populations showed a positive shift. (Figure S5A,B). These findings suggest that TALDO1 may be more closely associated with the transcriptional state of TREM2^+^ macrophages rather than broadly affecting all myeloid cell states. In addition, mIF staining was performed on HCC tumour tissues and paired adjacent non‐tumorous tissues. Compared with adjacent non‐tumorous tissues, HCC tumour tissues showed markedly stronger TALDO1, CD68 and CD206 signals. Further analysis revealed colocalization of TALDO1 with CD68^+^CD206^+^ macrophages, and Manders colocalization analysis showed significantly greater overlap in tumour tissues than in adjacent tissues. In parallel, the proportion of TALDO1^+^CD68^+^CD206^+^ cells was significantly increased in tumour tissues. These findings further support an association between TALDO1 and an M2‐like macrophage‐associated immunosuppressive microenvironment (Figure 6K).

To further validate these findings, we established a Transwell indirect co‐culture system using Huh7 cells and PMA induced THP‐1 derived macrophage like cells. Flow cytometric analysis showed that, compared with the control group, THP‐1 derived macrophage like cells co cultured with TALDO1 silenced Huh7 cells showed a significantly lower proportion of CD11b^+^CD206^+^ cells and a corresponding increase in CD11b^+^CD86^+^ cells (Figure 6L). These findings further support the association between TALDO1 and an immunosuppressive state characterized by M2 like macrophage features in HCC.

### 3.7 TALDO1 is associated with T cell exhaustion and Treg mediated immunosuppressive remodelling

**FIGURE 7:**
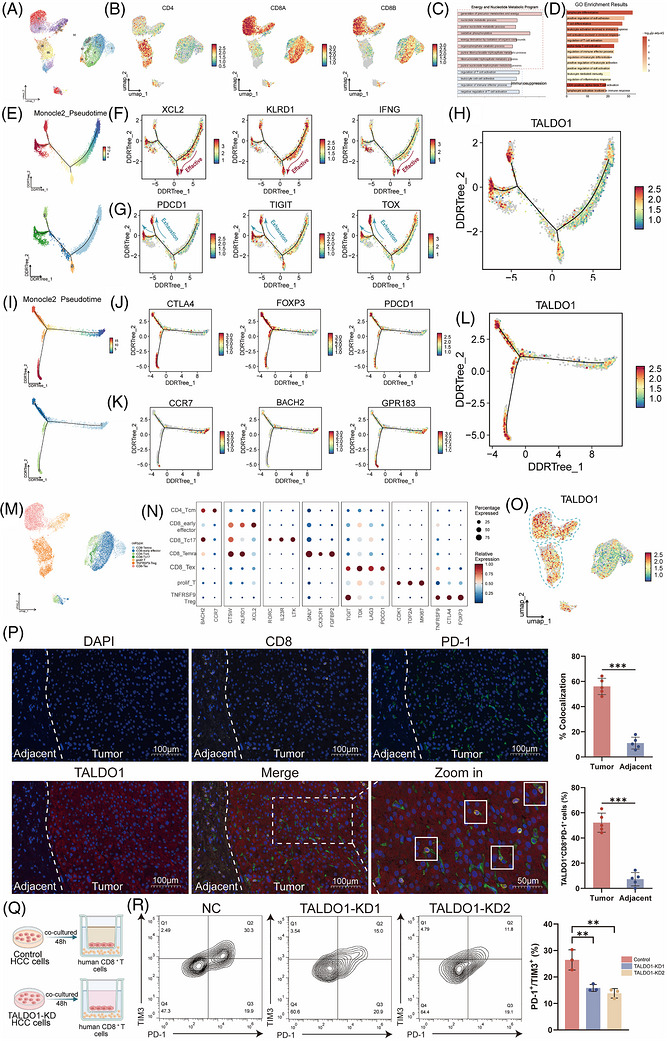
TALDO1 is associated with T cell exhaustion and Treg mediated immunosuppressive states. (A) Subclustering of T cells. (B) Identification of CD4^+^ T cells and CD8^+^ T cells. (C and D) Pathway enrichment analysis of T cells with high and low TALDO1 expression. (E) Pseudotime trajectory analysis of CD8^+^ T cells. (F and G) Functional state features of CD8^+^ T cells along the pseudotime trajectory. (H) Expression dynamics of TALDO1 along the pseudotime trajectory of CD8^+^ T cells. (I) Pseudotime trajectory analysis of CD4^+^ T cells. (J and K) Functional state features of CD4^+^ T cells along the pseudotime trajectory. (L) Expression dynamics of TALDO1 along the pseudotime trajectory of CD4^+^ T cells. (M and N) Identification of T cell types and expression of marker genes. (O) Expression of TALDO1 in T cell subsets. (P) mIF staining and quantitative analysis of TALDO1, CD8 and PD‐1 in HCC tumour tissues and paired adjacent tissues. Measurements from five randomly selected fields were averaged for each sample for statistical analysis (*n* = 5 per group). (Q) Schematic illustration of the Transwell indirect coculture system using Huh7 cells and human CD8^+^ T cells. (R) Flow cytometric analysis of PD‐1 and TIM‐3 expression in CD8^+^ T cells after coculture with control or TALDO1 knockdown Huh7 cells (*n* = 3 per group). *p < 0.05, **p < 0.01, and ***p < 0.001.

Given that TALDO1 expression was significantly positively correlated with Treg infiltration, we further investigated whether TALDO1 is associated with a T‐cell‐mediated immunosuppressive microenvironment. All T cells were subdivided into 25 clusters (Figure 7A) and classified into CD4^+^ T cells and CD8^+^ T cells based on marker genes (Figure 7B). T cells were then stratified into high and low TALDO1 expression groups, followed by pathway enrichment analysis. The results showed that energy metabolism‐ and immunosuppression‐related pathways were significantly activated in T cells with high TALDO1 expression (Figure 7C), whereas the TALDO1 low‐expression group exhibited prominent T‐cell activation‐related features (Figure 7D).

Pseudotime trajectory analysis was further performed for CD8^+^ T cells (Figure 7E), revealing two distinct functional state trajectories along differentiation, characterized by exhaustion‐related features and effector‐related features, respectively (Figure 7F,G). Notably, TALDO1 was highly expressed along the exhaustion‐associated trajectory, suggesting that TALDO1 may be associated with the exhausted state of CD8^+^ T cells (Figure 7H). In parallel, pseudotime trajectory analysis of CD4^+^ T cells was conducted (Figure 7I), in which the trajectory origin exhibited naïve T‐cell‐related features, while the terminal state gradually enriched for Treg‐associated features (Figure 7J,K). Consistently, TALDO1 was significantly enriched at the terminal stage of this trajectory (Figure 7L), further suggesting an association between TALDO1 and Treg‐related immunosuppressive states.

Meanwhile, based on specific marker genes, seven major T‐cell types were identified (Figure 7 M,N). The results showed that TALDO1 was highly expressed in TNFRSF9^+^ Treg cells and CD8^+^ exhausted T cells (Figure 7O). To further validate the association between TALDO1 and immunosuppressive T cell states, mIF staining was performed on HCC tumour tissues and paired adjacent tissues. Representative images showed increased TALDO1 signals in tumour tissues, with colocalization observed between TALDO1 and CD8^+^PD‐1^+^ cells. Manders colocalization analysis showed that the colocalization level of TALDO1 with CD8^+^PD‐1^+^ cells was higher in tumour tissues than in adjacent tissues. In addition, the proportion of TALDO1^+^CD8^+^PD 1^+^ cells was increased in tumour tissues (Figure 7P). These findings further support the association between TALDO1 and a T cell mediated immunosuppressive microenvironment.

To further validate this association, we established a Transwell indirect coculture system using Huh7 cells and human CD8^+^ T cells (Figure 7Q). Flow cytometric analysis showed that, compared with the control group, the proportion of PD‐1^+^TIM 3^+^ cells was reduced among CD8^+^ T cells cocultured with TALDO1 knockdown HCC cells (Figure 7R). These results suggest that reduced TALDO1 expression in HCC cells attenuates their ability to promote the acquisition of an exhaustion related phenotype in CD8^+^ T cells.

### 3.8 TALDO1 promotes HCC progression and facilitates the formation of an immunosuppressive tumour microenvironment

**FIGURE 8:**
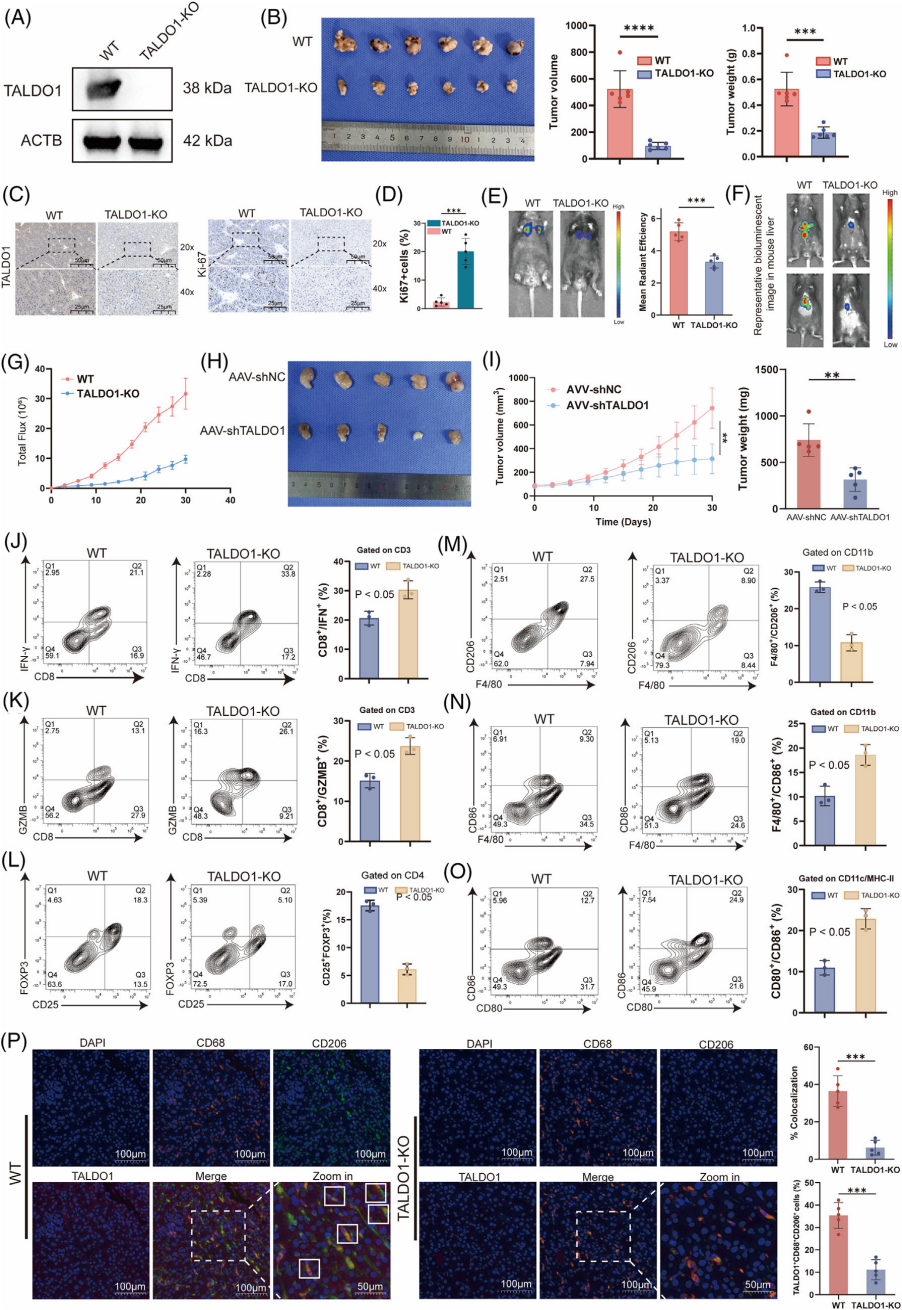
TALDO1 promotes HCC progression and the formation of an immunosuppressive microenvironment. (A) Validation of TALDO1 knockout efficiency. (B) Representative images of subcutaneous tumours and terminal tumour weight (*n* = 6 per group). (C) Representative images of Ki‐67 staining in tumour tissues. (D) Quantitative comparison of the proportion of Ki‐67‐positive cells in tumour tissues between the WT and TALDO1‐KO groups (*n* = 5 per group). (E) In vivo fluorescence imaging of the experimental lung metastasis model (*n* = 5 per group). (F and G) Representative bioluminescence images and total flux of orthotopic liver tumours (*n* = 5 per group). (H) Representative images of patient‐derived HCC xenografts treated with AAV‐shNC or AAV‐shTALDO1 (*n* = 5 per group). (I) Tumour growth curves and endpoint tumour weights of PDX treated with AAV‐shNC or AAV‐shTALDO1. (J and K) Proportions of CD8^+^IFN‐γ^+^ and CD8^+^GZMB^+^ T cells in orthotopic tumours (*n* = 3 per group). (L) Proportion of CD25^+^FOXP3^+^ Treg cells in orthotopic tumours (*n* = 3 per group). (M and N) Proportions of M2‐like macrophages and M1‐like macrophages in orthotopic tumours (*n* = 3 per group). (O) Proportion of CD80^+^CD86^+^ cells within the CD11c^+^MHC‐II^+^cell population (*n* = 3 per group). (P) mIF images of TALDO1, CD68, and CD206 staining in tumour tissues from the WT group and TALDO1‐KO group (*n* = 5 per group). *p < 0.05, **p < 0.01, and ***p < 0.001.

To further validate the role of TALDO1 in HCC progression, we generated a TALDO1‐KO Hepa1‐6 cell line (Figure 8A). TALDO1‐KO or control Hepa1‐6 cells were subcutaneously implanted into C57BL/6 mice. Representative tumour images showed that, compared with the control group, TALDO1 knockout significantly suppressed tumour growth, accompanied by a marked reduction in terminal tumour weight (Figure 8B). Ki‐67 staining further showed that the number of proliferating cells was significantly decreased in the TALDO1‐KO group compared with the WT group (Figure 8C,D). Subsequently, an experimental lung metastasis model was established in C57BL/6 mice by tail vein injection of luciferase‐labelled Luc‐Hepa1‐6 cells. In vivo imaging demonstrated that, compared with the WT group, the overall pulmonary metastatic fluorescence intensity was significantly reduced in the TALDO1‐KO group (Figure 8E), suggesting that TALDO1 knockout markedly impaired the lung metastatic capacity of HCC cells. Furthermore, an orthotopic liver implantation model was established by injecting TALDO1‐KO or WT Hepa1‐6 cells into the livers of C57BL/6 mice. In vivo fluorescence imaging showed that orthotopic tumours in the TALDO1‐KO group exhibited weaker fluorescence signals (Figure 8F,G). Collectively, these in vivo findings indicate that TALDO1 promotes tumour growth and metastasis in HCC. To further validate the role of TALDO1 in a human HCC model, we established a patient‐derived xenograft (PDX) model and treated mice with AAV‐shNC or AAV‐shTALDO1. Representative tumour images showed that PDX tumours in the AAV‐shTALDO1 group were markedly smaller than those in the AAV‐shNC group (Figure 8H). In addition, AAV‐mediated TALDO1 knockdown significantly inhibited PDX tumour growth and reduced tumour weight at the experimental endpoint (Figure 8I). These results further indicate that TALDO1 silencing suppresses the growth of patient‐derived HCC tumours, supporting a tumour‐promoting role for TALDO1 in HCC progression. To preliminarily assess the potential druggability of TALDO1, cMap/L1000CDS2 analysis identified several candidate compounds, such as narciclasine, emetine, and A‐443654. Molecular docking analysis showed that narciclasine and A‐443654 exhibited favourable predicted binding energies with TALDO1 and were predicted to occupy the substrate binding pocket near Lys142 (Figure S5D).

To more comprehensively evaluate the effects of TALDO1 on the TME, we performed flow cytometric analysis of orthotopic liver tumour tissues from the TALDO1‐KO and WT groups. Cells were first gated on CD45^+^ cells, followed by assessment of CD8, IFN‐γ, and GZMB expression within the CD3^+^ T‐cell population. Compared with the WT group, the proportions of CD8^+^IFN‐γ^+^ and CD8^+^GZMB^+^ T cells were significantly increased in the TALDO1‐KO group (Figure 8J,K), indicating enhanced effector function of CD8^+^ T cells following TALDO1 knockout. Cells were then sequentially gated on CD45^+^, CD3^+^, and CD4^+^ populations, followed by identification of CD25^+^FOXP3^+^ Treg cells. The proportion of Treg cells in tumour tissues was reduced in the TALDO1‐KO group (Figure 8L). Meanwhile, F4/80, CD206 and CD86 expression was assessed within the CD45^+^CD11b^+^ myeloid cell population. Compared with the WT group, TALDO1 knockout reduced the proportion of F4/80^+^CD206^+^ M2‐like macrophages and increased the proportion of F4/80^+^CD86^+^ M1‐like macrophages (Figure 8 M,N). In addition, within the CD11c^+^MHC‐II^+^ dendritic cell population, the proportion of CD80^+^CD86^+^ cells was significantly increased in the TALDO1‐KO group (Figure 8O), suggesting enhanced dendritic cell activation. We further performed mIF staining of tumour tissues from the TALDO1‐KO and WT groups. The results showed that the expression levels of TALDO1, CD68 and CD206 were markedly higher in the WT group than in the TALDO1‐KO group (Figure 8P). Taken together, these findings further indicate that TALDO1 contributes to the formation of an immunosuppressive microenvironment.

## 4 DISCUSSION

HCC is characterized by marked metabolic heterogeneity and a complex immunosuppressive TME, both of which are closely associated with tumour progression, therapeutic response and patient prognosis.^9^, ^25^, ^26^ However, the key factors driving metabolic reprogramming in HCC and shaping the immunosuppressive microenvironment remain incompletely understood. Our findings indicate that TALDO1 participates in tumour cell metabolic reprogramming and may also contribute to the establishment of an immunosuppressive TME, suggesting that it could represent a potential therapeutic target in HCC.

In this study, we first used a deep learning approach to extract latent metabolism related features from high dimensional omics data and identified TALDO1 as a candidate gene that may drive metabolic dysregulation in HCC. Subsequent analyses across multiple cohorts, together with immunohistochemical evaluation of postoperative HCC specimens, further showed that TALDO1 was consistently overexpressed in tumour tissues and was significantly associated with poor prognosis. Mouse models further demonstrated that TALDO1 deficiency markedly suppressed tumour growth and metastasis. Notably, in a PDX model, AAV mediated TALDO1 silencing similarly inhibited tumour growth and reduced tumour weight at the endpoint. These findings support a tumour promoting role for TALDO1 in patient derived HCC and strengthen its potential clinical relevance as a therapeutic target.

To further investigate the role of TALDO1 in HCC metabolism, we performed RNA sequencing and found that genes downregulated following TALDO1 silencing were significantly enriched in pathways related to lipid metabolism. Subsequent experiments confirmed that TALDO1 knockdown reduced the expression of lipogenic genes, and decreased lipid droplet accumulation. Previous studies have mainly focused on the role of TALDO1 in nucleotide biosynthesis and redox homeostasis, whereas its contribution to lipid metabolism and the underlying mechanisms remain unclear.^20^, ^27^, ^28^ Notably, TALDO1 knockdown did not significantly alter the NADPH/NADP^+^ ratio, and supplementation with acetate or citrate failed to restore lipid accumulation or lipogenic gene expression. These findings suggest that the inhibitory effect of TALDO1 loss on lipogenesis cannot be explained solely by altered NADPH homeostasis or limited carbon substrate availability. Given that TALDO1 knockdown suppressed lipogenesis in our study, we further explored the potential molecular basis of this effect. TALDO1 knockdown selectively reduced SREBP1 transcript levels and the abundance of mSREBP1 protein. Re‐expression of SREBP1 restored the reduced expression of lipogenic enzymes caused by TALDO1 loss and partially reversed the decrease in cellular lipid levels. These results suggest that SREBP1 is involved in TALDO1 mediated regulation of lipid metabolism. Notably, RNA‐seq enrichment analysis indicated activation of AMPK signalling following TALDO1 silencing. TALDO1 is a key enzyme in the nonoxidative branch of the pentose phosphate pathway, and its dysregulation may lead to metabolic imbalance and cellular stress.^29^, ^30^ As an important energy sensor, AMPK can be activated by energy stress and redox imbalance.^31^, ^32^ We therefore investigated whether AMPK signalling contributes to TALDO1 related alterations in lipid metabolism. TALDO1 knockdown increased p‐AMPK levels and was accompanied by reduced mSREBP1 expression and lower levels of lipogenic enzymes. Given that SREBP1 is a key transcriptional regulator of lipogenesis, we further performed rescue and pharmacological intervention experiments. TALDO1 re expression reversed these changes, whereas treatment with the AMPK inhibitor Compound C partially restored the expression of mSREBP1 and lipogenesis related proteins. Collectively, these findings suggest that TALDO1 may influence SREBP1 maturation and downstream lipogenic programs by modulating AMPK activity. They also support the involvement of the AMPK/SREBP1 axis in TALDO1 mediated regulation of lipid synthesis. Metabolic dysregulation affects not only tumour cells themselves but can also remodel the TME through metabolite secretion, nutrient competition, and intercellular signalling.^33^, ^34^ On this basis, we further examined the effects of TALDO1 silencing on lipid release by HCC cells. We found reduced levels of multiple fatty acids and lipid mediators. Some of these lipid mediators, including PGE2, have been reported to promote an immunosuppressive macrophage phenotype and impair the effector function of CD8^+^ T cells.^35^, ^36^ These findings suggest that TALDO1 may contribute to remodelling of the immune microenvironment in HCC through its regulation of lipid metabolism.

We next investigated whether TALDO1 is involved in the development of an immunosuppressive TME. scRNA‐seq analyses of multiple resected HCC specimens showed that TALDO1 expression was enriched in M2 like macrophages and Treg cells. In CD8^+^ T cells, TALDO1 expression was higher in the exhausted subset and gradually increased along the exhaustion pseudotime trajectory. Similarly, in CD4^+^ T cells, TALDO1 expression increased progressively along the trajectory toward Treg differentiation. In addition, in silico perturbation using Geneformer showed that deletion of the TALDO1 token predicted a negative state shift in TREM2^+^ macrophages, whereas other myeloid cell populations did not show a comparable pattern. This finding suggests that TALDO1 may be associated with maintenance of the TREM2^+^ macrophage state. mIF staining further supported these observations. Compared with adjacent tissues, tumour tissues showed greater colocalization of TALDO1 with CD68^+^CD206^+^ cells and CD8^+^PD‐1^+^ cells. The proportions of TALDO1^+^CD68^+^CD206^+^ cells and TALDO1^+^CD8^+^PD‐1^+^ cells were also markedly increased. Using coculture systems comprising HCC cells with THP‐1 derived macrophage like cells or human CD8^+^ T cells, we further found that TALDO1 silencing weakened the ability of HCC cells to induce M2 like macrophage features and promote exhaustion associated phenotypes. Together, these findings support a close association between TALDO1 and an immunosuppressive TME. This association was further supported in an orthotopic HCC model. Compared with the WT group, tumours in the TALDO1‐KO group showed reduced proportions of Treg cells and M2 like macrophages, together with increased proportions of effector T cells, M1 like macrophages, and activated dendritic cells. However, because TALDO1 loss can suppress tumour cell proliferation, changes in immune cell infiltration in orthotopic tumours may be secondary to reduced tumour burden and altered tumour cell states. Meanwhile, changes in immune cell composition may further affect antitumour responses and tumour progression. Nevertheless, both tumour cell growth suppression and immune microenvironment remodelling may contribute to the tumour suppressive effects induced by TALDO1 loss. Their respective contributions and interrelationships require further investigation.

This study further expands the understanding of the role of TALDO1 in HCC and suggests that targeting TALDO1 may represent a promising therapeutic strategy. Notably, a recent study identified AO‐022 as an allosteric inhibitor of TALDO1 and provided consistent antitumour evidence in breast cancer across in vitro experiments, animal models and patient derived organoids.^37^ These findings offer preliminary support for the pharmacological tractability of TALDO1 and further underscore its potential as a therapeutic target. However, AO‐022 has so far been evaluated primarily in breast cancer. Whether pharmacological inhibition of TALDO1 can ameliorate metabolic alterations and immunosuppression related phenotypes in HCC remains to be determined.

This study has several limitations. First, although our findings support the involvement of AMPK/SREBP1 signalling in TALDO1‐mediated regulation of lipogenesis, the upstream metabolic basis by which TALDO1 regulates AMPK activation remains unclear and requires further investigation. Second, Geneformer based in silico perturbation provides only computational predictions of cell state changes. As this model was not trained on HCC or other tumour datasets, its simulation of tumour associated myeloid cells may be subject to bias. Therefore, the Geneformer derived findings require further validation through CRISPR mediated perturbation in human myeloid cells. Third, this study primarily evaluated the impact of tumour cell intrinsic TALDO1 loss on the immune microenvironment. Although the coculture experiments further support a role for tumour cell TALDO1 in remodelling immune states, single cell analyses showed that TALDO1 is also expressed in myeloid cells and T cells. Thus, a direct role of TALDO1 within immune cells cannot be excluded. Future studies using myeloid cell specific or T cell specific conditional knockout models will be needed to further define the function of TALDO1 in immune cells.

In summary, our study demonstrates that TALDO1 not only promotes lipid metabolic reprogramming in HCC cells but also participates in the establishment of an immunosuppressive TME. These findings suggest that TALDO1 may represent a key molecule linking tumour metabolic dysregulation to microenvironmental remodelling and may serve as a potential target for precision therapy in HCC.

## AUTHOR CONTRIBUTIONS

**Fenglin Lv**: Conceptualization; data curation; formal analysis; investigation; methodology; project administration; resources; software; validation; visualization; writing—original draft. **Huaxin Zhou**: Conceptualization; data curation; formal analysis; investigation; methodology; project administration; validation; visualization; writing—original draft. **Jingyan Yang**: Conceptualization; data curation; formal analysis; investigation; methodology; project administration; validation; visualization; writing—original draft. **Jianglei Xu**: Conceptualization; data curation; formal analysis. **Fanqing Bi**: Validation; visualization. **Yi Zhou**: Validation; visualization. **Hao Zhang**: Project administration; supervision; conceptualization; data curation; formal analysis; investigation; methodology; writing—review and editing. **Qian Ye**: Project administration; supervision; conceptualization; data curation; formal analysis; investigation; methodology; writing—review and editing. **Lin Gao**: Project administration; supervision; conceptualization; data curation; formal analysis; investigation; methodology; writing—review and editing. **Bin Jin**: Project administration; supervision; conceptualization; data curation; formal analysis; investigation; methodology; writing—review and editing.

## CONFLICT OF INTEREST STATEMENT

The authors declare no conflicts of interest.

## ETHICS STATEMENT AND CONSENT

The study followed the Declaration of Helsinki. The Animal Use and Care Committee and the Scientific Research Ethics Committee of the Second QILU Hospital of Shandong University authorized animal experiments as well as the use of human samples and data (License No. KYLL202509708).

## Supporting information



Supporting Information

Supporting Information

Supporting Information

Supporting Information

Supporting Information

Supporting Information

Supporting Information

Supporting Information

Supporting Information

Supporting Information

Supporting Information

## Data Availability

The single‐cell RNA sequencing data generated in this study have been deposited in the Genome Sequence Archive for Human (GSA‐Human) of the National Genomics Data Center (NGDC), China National Center for Bioinformation, Chinese Academy of Sciences, under accession number HRA016357. All data and original code used in this manuscript are available from the corresponding author upon reasonable request.

## References

[1] Zhang C‐H , Cheng Y , Zhang S , Fan J , Gao Q . Changing epidemiology of hepatocellular carcinoma in Asia. Liver Int. 2022;42(9):2029–2041. doi:10.1111/liv.15251 35319165

[2] Mcglynn KA , Petrick JL , El‐Serag HB . Epidemiology of hepatocellular carcinoma. Hepatology. 2021;73(1):4–13. doi:10.1002/hep.31288 PMC7577946 32319693

[3] Fu Y , Maccioni L , Wang XW , Greten TF , Gao B . Alcohol‐associated liver cancer. Hepatology. 2024;80(6):1462–1479. doi:10.1097/HEP.0000000000000890 38607725

[4] Rumgay H , Arnold M , Ferlay J , et al. Global burden of primary liver cancer in 2020 and predictions to 2040. J Hepatol. 2022;77(6):1598–1606. doi:10.1016/j.jhep.2022.08.021 36208844 PMC9670241

[5] Toh MR , Wong EYT , Wong SH , et al. Global epidemiology and genetics of hepatocellular carcinoma. Gastroenterology. 2023;164(5):766–782. doi:10.1053/j.gastro.2023.01.033 36738977

[6] Sies H , Mailloux RJ , Jakob U . Fundamentals of redox regulation in biology. Nat Rev Mol Cell Biol. 2024;25(9):701–719. doi:10.1038/s41580-024-00730-2 38689066 PMC11921270

[7] Faubert B , Solmonson A , DeBerardinis RJ . Metabolic reprogramming and cancer progression. Science. 2020(6487):368:eaaw5473. 32273439 10.1126/science.aaw5473 PMC7227780

[8] Park S , Hall MN . Metabolic reprogramming in hepatocellular carcinoma: mechanisms and therapeutic implications. Exp Mol Med. 2025;57(3):515–523. doi:10.1038/s12276-025-01415-2 40025169 PMC11958682

[9] Yang F , Hilakivi‐Clarke L , Shaha A , et al. Metabolic reprogramming and its clinical implication for liver cancer. Hepatology. 2023;78(5):1602–1624. 36626639 10.1097/HEP.0000000000000005 PMC10315435

[10] Cassim S , Raymond V‐A , Dehbidi‐Assadzadeh L , Lapierre P , Bilodeau M . Metabolic reprogramming enables hepatocarcinoma cells to efficiently adapt and survive to a nutrient‐restricted microenvironment. Cell Cycle. 2018;17(7):903–916. doi:10.1080/15384101.2018.1460023 29633904 PMC6056217

[11] Khalaf K , Hana D , Chou JT‐T , Singh C , Mackiewicz A , Kaczmarek M . Aspects of the tumor microenvironment involved in immune resistance and drug resistance. Front Immunol. 2021;12:656364. doi:10.3389/fimmu.2021.656364 34122412 PMC8190405

[12] Saravia J , Chi H . Immunometabolism of regulatory T cells in cancer. Oncogene. 2025;44(25):2011–2024. doi:10.1038/s41388-025-03458-1 40468052 PMC12167712

[13] Yan Y , Huang L , Liu Y , et al. Metabolic profiles of regulatory T cells and their adaptations to the tumor microenvironment: implications for antitumor immunity. J Hematol Oncol. 2022;15(1):104. doi:10.1186/s13045-022-01322-3 35948909 PMC9364625

[14] Kondo M , Kumagai S , Nishikawa H . Metabolic advantages of regulatory T cells dictated by cancer cells. Int Immunol. 2024;36(2):75–86. doi:10.1093/intimm/dxad035 37837615

[15] Li M , Yang Y , Xiong L , Jiang P , Wang J , Li C . Metabolism, metabolites, and macrophages in cancer. J Hematol Oncol. 2023;16(1):80. doi:10.1186/s13045-023-01478-6 37491279 PMC10367370

[16] Doncevic D , Herrmann C . Biologically informed variational autoencoders allow predictive modeling of genetic and drug‐induced perturbations. Bioinformatics. 2023;39(6):btad387. doi:10.1093/bioinformatics/btad387 37326971 PMC10301695

[17] Stincone A , Prigione A , Cramer T , et al. The return of metabolism: biochemistry and physiology of the pentose phosphate pathway. Biol Rev. 2015;90(3):927–963. doi:10.1111/brv.12140 25243985 PMC4470864

[18] Qian Y , Banerjee S , Grossman CE , et al. Transaldolase deficiency influences the pentose phosphate pathway, mitochondrial homoeostasis and apoptosis signal processing. Biochem J. 2008;415(1):123–134. doi:10.1042/BJ20080722 18498245

[19] Jiang P , Du W , Wu M . Regulation of the pentose phosphate pathway in cancer. Protein & Cell. 2014;5(8):592–602. doi:10.1007/s13238-014-0082-8 25015087 PMC4112277

[20] Ding Y , Gong C , Huang D , et al. Synthetic lethality between HER2 and transaldolase in intrinsically resistant HER2‐positive breast cancers. Nat Commun. 2018;9(1):4274. doi:10.1038/s41467-018-06651-x 30323337 PMC6189078

[21] Cen K , Lu C . Prognostic and immune infiltration analysis of Transaldolase 1 (TALDO1) in hepatocellular carcinoma. Int J Gen Med. 2023;16:5779–5788. doi:10.2147/IJGM.S425490 38089714 PMC10715008

[22] Shi X , Zhao Y , Li K , et al. Plasma proteomic signature for preoperative prediction of microvascular invasion in HCC. JHEP Reports. 2025;7(9):101481. doi:10.1016/j.jhepr.2025.101481 40823177 PMC12355497

[23] Herzig S , Shaw RJ . AMPK: guardian of metabolism and mitochondrial homeostasis. Nat Rev Mol Cell Biol. 2018;19(2):121–135. doi:10.1038/nrm.2017.95 28974774 PMC5780224

[24] Arner EN , Rathmell JC . Metabolic programming and immune suppression in the tumor microenvironment. Cancer Cell. 2023;41(3):421–433. doi:10.1016/j.ccell.2023.01.009 36801000 PMC10023409

[25] Lin J , Rao D , Zhang M , Gao Q . Metabolic reprogramming in the tumor microenvironment of liver cancer. J Hematol Oncol. 2024;17(1):6. doi:10.1186/s13045-024-01527-8 38297372 PMC10832230

[26] Xu F‐Q , Dong M‐M , Wang Z‐F , Cao L‐D . Metabolic rearrangements and intratumoral heterogeneity for immune response in hepatocellular carcinoma. Front Immunol. 2023;14:1083069. doi:10.3389/fimmu.2023.1083069 36776894 PMC9908004

[27] Perl A , Hanczko R , Telarico T , Oaks Z , Landas S . Oxidative stress, inflammation and carcinogenesis are controlled through the pentose phosphate pathway by transaldolase. Trends Mol Med. 2011;17(7):395–403. doi:10.1016/j.molmed.2011.01.014 21376665 PMC3116035

[28] Moriyama T , Tanaka S , Nakayama Y , et al. Two isoforms of TALDO1 generated by alternative translational initiation show differential nucleocytoplasmic distribution to regulate the global metabolic network. Sci Rep. 2016;6:34648. doi:10.1038/srep34648 27703206 PMC5050407

[29] Hanczko R , Fernandez DR , Doherty E , et al. Prevention of hepatocarcinogenesis and increased susceptibility to acetaminophen‐induced liver failure in transaldolase‐deficient mice by N‐acetylcysteine. J Clin Invest. 2009;119(6):1546–1557. doi:10.1172/JCI35722 19436114 PMC2689120

[30] Perl A , Qian Y , Chohan KR , et al. Transaldolase is essential for maintenance of the mitochondrial transmembrane potential and fertility of spermatozoa. Proc Natl Acad Sci. 2006;103(40):14813–14818. doi:10.1073/pnas.0602678103 17003133 PMC1595434

[31] Hardie DG , Ross FA , Hawley SA . AMPK: a nutrient and energy sensor that maintains energy homeostasis. Nat Rev Mol Cell Biol. 2012;13(4):251–262. doi:10.1038/nrm3311 22436748 PMC5726489

[32] Auciello FR , Ross FA , Ikematsu N , Hardie DG . Oxidative stress activates AMPK in cultured cells primarily by increasing cellular AMP and/or ADP. FEBS Lett. 2014;588(18):3361–3366. doi:10.1016/j.febslet.2014.07.025 25084564 PMC4158911

[33] Lyssiotis CA , Kimmelman AC . Metabolic interactions in the tumor microenvironment. Trends Cell Biol. 2017;27(11):863–875. doi:10.1016/j.tcb.2017.06.003 28734735 PMC5814137

[34] Chang C‐H , Qiu J , O'Sullivan D , et al. Metabolic competition in the tumor microenvironment is a driver of cancer progression. Cell. 2015;162(6):1229–1241. doi:10.1016/j.cell.2015.08.016 26321679 PMC4864363

[35] Mazzoni M , Mauro G , Erreni M , et al. Senescent thyrocytes and thyroid tumor cells induce M2‐like macrophage polarization of human monocytes via a PGE2‐dependent mechanism. J Exp Clin Cancer Res. 2019;38(1):208. doi:10.1186/s13046-019-1198-8 31113465 PMC6528237

[36] Lacher SB , Dörr J , De Almeida GP , et al. PGE2 limits effector expansion of tumour‐infiltrating stem‐like CD8+ T cells. Nature. 2024;629(8011):417–425. doi:10.1038/s41586-024-07254-x 38658748 PMC11078747

[37] Xu G , Huang R , Wumaier R , et al. Proteomic profiling of serum extracellular vesicles identifies diagnostic signatures and therapeutic targets in breast cancer. Cancer Res. 2024;84(19):3267–3285. doi:10.1158/0008-5472.CAN-23-3998 38900939 PMC11443238

