# Supplementary material for: TALDO1 promotes lipid metabolic reprogramming and immunosuppressive microenvironment remodelling in hepatocellular carcinoma
Source: Clin Transl Med. 2026 Aug 10;16(8):e70771. doi: 10.1002/ctm2.70771 (PMC13458028; doi:10.1002/ctm2.70771)
Supplement: Supplementary file 1 [file CTM2-16-e70771-s006.docx]

**Figure S1. Feature contribution in the XGBoost model and survival analysis of ATP6V1C1. A.** Representative decision tree structure in the XGBoost risk model. **B.** Kaplan–Meier analysis of OS in patients with high and low ATP6V1C1 expression. **C.** Kaplan–Meier analysis of DFS in patients with high and low ATP6V1C1 expression.

**Figure S2. TALDO1 expression across HCC etiologies and metabolic effects of TALDO1 silencing. A.** TALDO1 expression in HBV related HCC and normal liver tissues from GSE121248, and in NAFLD related HCC and normal liver tissues from GSE164441. **B.** NADPH/NADP⁺ ratios in TALDO1-knockdown Huh7 and Hep3B cells. **C.** Effects of acetate or citrate supplementation on triglyceride, free fatty acid, and polyunsaturated fatty acid levels in TALDO1-knockdown cells. **D.** Effects of acetate or citrate supplementation on FASN, ACC1, and SCD1 mRNA expression in TALDO1-knockdown cells.

**Figure S3. Effects of the AMPK inhibitor on lipid metabolism in TALDO1 knockdown cells and Harmony batch correction results. A.** Effects of Compound C on triglyceride, free fatty acid, and polyunsaturated fatty acid levels in Huh7 and Hep3B cells with TALDO1 knockdown. **B–C.** Effects of Compound C on the mRNA expression of lipogenic enzymes in Huh7 (B) and Hep3B (C) cells with TALDO1 knockdown. **D.** UMAP distribution of the single cell dataset before and after Harmony correction. **E.** UMAP distribution of major cell types before and after Harmony correction.

**Figure S4. Spatial transcriptomic analysis and validation of TALDO1⁺TREM2⁺ macrophages in an independent single-cell cohort. A.** Spatial distribution of clusters across the spatial transcriptomic sections. **B.** Dot plot of marker genes used for cell type annotation of spatial transcriptomic clusters. **C.** Comparison of normalized distances from TALDO1-high and TALDO1-low TAMs to the nearest tumor boundary. **D.** UMAP visualization of major cell types in the GSE149614 cohort. **E.** UMAP visualization of myeloid cell subsets in the GSE149614 cohort. **F.** Dot plot of representative marker genes for myeloid cell subsets. **G.** Relative proportions of myeloid cell subsets in normal liver and HCC tissues.

**Figure S5. Geneformer based TALDO1 perturbation analysis, immune association analysis, and candidate drug prediction. A.** Predicted direction and magnitude of cell state changes across myeloid cell subtypes after simulated deletion of the TALDO1 token using Geneformer. Dot color indicates directional change, dot size indicates the magnitude of change, and the bar plot on the right shows the number of cells included in the analysis. **B.** UMAP visualization of predicted directional state changes in myeloid cells after TALDO1 token deletion. **C.** Spearman correlation analysis between TALDO1 expression and immune checkpoint related genes as well as immune signature scores. **D.** Candidate compounds predicted by cMap to reverse the TALDO1 related transcriptional signature and molecular docking results between candidate compounds and TALDO1. The left panel shows Vina docking scores, the middle panel shows distances between candidate compounds and TALDO1 binding site residues, and the right panel shows cMap reversal scores.
